# Supplementary material for: Quality of life following a lower limb reconstructive procedure: a protocol for the development of a conceptual framework
Source: BMJ Open. 2020 Dec 10;10(12):e040378. doi: 10.1136/bmjopen-2020-040378 (PMC7733194; doi:10.1136/bmjopen-2020-040378)
Supplement: Supplementary data [file bmjopen-2020-040378supp001.pdf]

Database: Ovid MEDLINE(R) ALL <1946 to August 16, 2019>

19/08/2019

DRAFT Search Strategy:

----

1 Ankle Fractures/ (1253)

2 Femoral Fractures/ (15922)

3 Tibial Fractures/ (14766)

4 1 or 2 or 3 (29498)

5 ((fractur$ or trauma$) adj3 (lower extremit$ or lower limb$)).ti,ab. (2992)

6 ((fractur$ or trauma$) adj3 (leg or legs or thigh$ or knee or knees or shin or shins or shinbone$ or foot or midfoot or hindfoot or feet or ankle or ankles or anklebone$ or pilon or heel or heels or heelbone$ or toe or toes)).ti,ab. (9355)

7 ((fractur$ or trauma$) adj3 (femur$ or femoral or tibia$ or fibula$ or patella$ or kneecap$ or knee cap$)).ti,ab. (33008)

8 ((fractur$ or trauma$) adj3 (metatarsal$ or metatarsus or metatarsi or tarsus or tarsi or tarsal or tarsals or cuneiform or cuboid or navicular or calcane$ or talus or astragalus or tali or bimalleol$ or malleol$ or trimalleol$)).ti,ab. (5249)

9 ((Pott$ or Cotton$) adj fractur$).ti,ab. (30)

10 or/5-9 (46979)

11 lower extremity/ or exp foot/ or knee/ or leg/ or thigh/ (146797)

12 leg injuries/ or ankle injuries/ or foot injuries/ or knee injuries/ (38409)

13 exp Leg Bones/ (92594)

14 exp Foot Bones/ (17063)

15 11 or 12 or 13 or 14 (273200)

16 (lower extremit$ or lower limb$).ti,ab. (94764)

17 (leg or legs or thigh$ or knee or knees or shin or shins or shinbone$ or foot or midfoot or hindfoot or feet or ankle or ankles or anklebone$ or pilon or heel or heels or heelbone$ or toe or toes).ti,ab. (396803)

18 (femur$ or femoral or tibia$ or fibula$ or patella$ or kneecap$ or knee cap$).ti,ab. (237589)

19 (metatarsal$ or metatarsus or metatarsi or tarsus or tarsi or tarsal or tarsals or cuneiform or cuboid or navicular or calcane$ or talus or astragalus or tali or bimalleol$ or malleol$ or trimalleol$).ti,ab. (36596)

20 or/15-19 (717215)

21 Fractures, Bone/ (62396)

22 20 and 21 (18698)

23 (fractur$ or trauma$).ti,ab. (546587)

24 15 and 23 (39512)

25 exp Fracture Dislocation/ (2087)

26 Fractures, Avulsion/ (117)

27 Fractures, Closed/ (3275)

28 Fractures, Comminuted/ (2413)

29 Fractures, Compression/ (2080)

30 Fractures, Malunited/ (1551)

31 Fractures, Multiple/ (114)

32 Fractures, Open/ (5278)

33 Fractures, Stress/ (3216)

34 exp Fractures, Ununited/ (10494)

35 Intra-Articular Fractures/ (1104)

36 Periprosthetic Fractures/ (941)

37 Fracture Healing/ (12723)

38 Crush Injuries/ (121)

39 (crush$ adj2 (injur$ or trauma$ or fractur$)).ti,ab. (3676)

40 or/25-39 (43086)

41 20 and 40 (18153)

42 exp Multiple Trauma/ (12521)

43 (polytrauma$ or poly-trauma$ or multitrauma$ or multi-trauma$).ti,ab. (4627)

44 ((complex$ or complicat$ or severe$ or severity or serious or major or multiple) adj2 (fractur$ or trauma$)).ti,ab. (36622)

45 or/42-44 (48056)

46 20 and 45 (7375)

47 4 or 10 or 22 or 24 or 41 or 46 (87320)

48 ((bone$ or fracture$) adj3 (heal or heals or healed or healing)).ti,ab. (21408)

49 ((bone$ or fracture$) adj3 (union$ or nonunion$ or non union$ or ununite$)).ti,ab. (8182)

50 ((bone$ or fracture$) adj3 (malunion$ or mal union$ or deform$)).ti,ab. (4430)

51 (osteomyelitis or ((bone$ or fracture$) adj3 infect$)).ti,ab. (28503)

52 exp Osteomyelitis/ (22216)

53 or/48-52 (66572)

54 20 and 53 (21088)

55 ((femur$ or femoral or tibia$ or fibula$ or patella$ or kneecap$ or knee cap$) adj3 (heal or heals or healed or healing)).ti,ab. (1389)

56 ((metatarsal$ or metatarsus or metatarsi or tarsus or tarsi or tarsal or tarsals or cuneiform or cuboid or navicular or calcane$ or talus or astragalus or tali or bimalleol$ or malleol$ or trimalleol$) adj3 (heal or heals or healed or healing)).ti,ab. (107)

57 ((femur$ or femoral or tibia$ or fibula$ or patella$ or kneecap$ or knee cap$) adj3 (union$ or nonunion$ or non union$ or ununite$)).ti,ab. (1699)
58 ((metatarsal$ or metatarsus or metatarsi or tarsus or tarsi or tarsal or tarsals or cuneiform or cuboid or navicular or calcane$ or talus or astragalus or tali or bimalleol$ or malleol$ or trimalleol$) adj3 (union$ or nonunion$ or non union$ or ununite$)).ti,ab. (151)
59 ((femur$ or femoral or tibia$ or fibula$ or patella$ or kneecap$ or knee cap$) adj3 (malunion$ or mal union$ or deform$)).ti,ab. (1822)
60 ((metatarsal$ or metatarsus or metatarsi or tarsus or tarsi or tarsal or tarsals or cuneiform or cuboid or navicular or calcane$ or talus or astragalus or tali or bimalleol$ or malleol$ or trimalleol$) adj3 (malunion$ or mal union$ or deform$)).ti,ab. (528)
61 ((femur$ or femoral or tibia$ or fibula$ or patella$ or kneecap$ or knee cap$) adj3 (osteomyelitis or infect$)).ti,ab. (1622)
62 ((metatarsal$ or metatarsus or metatarsi or tarsus or tarsi or tarsal or tarsals or cuneiform or cuboid or navicular or calcane$ or talus or astragalus or tali or bimalleol$ or malleol$ or trimalleol$) adj3 (osteomyelitis or infect$)).ti,ab. (360)
63 or/55-62 (7267)
64 Limb Salvage/ (3799)
65 ((limb or limbs) adj3 (restor$ or reconstruct$ or salvag$)).ti,ab. (8371)
66 Ilizarov Technique/ (1280)
67 External Fixators/ (5652)
68 Orthopedic Fixation Devices/ (4991)
69 Fracture Fixation/ (18000)
70 Bone Lengthening/ (2175)
71 Ilizarov.ti,ab. (2107)
72 (external adj2 (fixat$ or frame$ or cage$)).ti,ab. (9626)
73 (circular adj2 (fixat$ or frame$ or cage$)).ti,ab. (609)
74 Taylor Spatial Frame$.ti,ab. (194)
75 TSF.ti,ab. (1209)
76 True Lok Hex.ti,ab. (0)
77 TLHex.ti,ab. (0)
78 or/64-77 (43999)
79 20 and 78 (20689)
80 47 or 54 or 63 or 79 (108579)
81 exp qualitative research/ (47998)
82 "Surveys and Questionnaires"/ (433413)
83 Self Report/ (27877)
84 exp Attitude/ (546190)

85 Focus Groups/ (27342)

86 Ethnology/ (1581)

87 discourse analysis.mp. (1718)

88 content analysis.mp. (25061)

89 ethnographic research.mp. (887)

90 ethnological research.mp. (7)

91 constant comparative method.mp. (1463)

92 qualitative validity.mp. (19)

93 purposive sample.mp. (3194)

94 observational method$.mp. (713)

95 field stud$.mp. (14522)

96 theoretical sampl$.mp. (635)

97 phenomenology.mp. (8689)

98 phenomenological research.mp. (431)

99 life experience$.mp. (5101)

100 or/81-99 (959292)

101 interview$.mp. or interviews/ or Interviews as Topic/ (365560)

102 qualitative.mp. (224736)

103 or/101-102 (512625)

104 100 or 103 (1295594)

105 ethnograph$.mp. (10148)

106 phenomenol$.mp. (24867)

107 grounded theory.mp. (10833)

108 (grounded adj (theor$ or study or studies or research or analys?s)).mp. (10995)

109 (emic or etic or hermeneutic$ or heuristic$ or semiotic$).mp. (16188)

110 (data adj1 saturat$).tw. (1054)

111 participant observ$.tw. (4226)

112 (action research or cooperative inquir$ or co operative inquir$ or co-operative inquir$).mp. (3997)

113 (field adj (study or studies or research or observation$)).tw. (18880)

114 theoretical sampl$.mp. (635)

115 (purpos$ adj4 sampl$).mp. (13410)

116 (focus adj group$).mp. (47371)

117 (account or accounts or unstructured or open-ended or open ended or text$ or narrative$).mp. (603046)

118 (life world or life-world or conversation analys?s or personal experience$ or theoretical saturation).mp. (14630)

119 lived experience$.tw. (5075)

120 (theme$ or thematic).mp. (97515)

121 (observational adj (method$ or research or stud$)).mp. (142328)

122 questionnaire$.mp. (691305)

123 content analysis.mp. (25061)

124 thematic analysis.mp. (15722)

125 discourse analys?s.mp. (1756)

126 ((discourse$ or discurs$) adj3 analys?s).tw. (2062)

127 (constant adj (comparative or comparison)).mp. (4283)

128 narrative analys?s.mp. (1157)

129 or/105-128 (1573717)

130 104 or 129 (2205208)

131 80 and 130 (5967)

132 survey$.ti,ab. (598311)

133 (mixed method$ or multimethod$ or multi-method$ or multi method$).mp. (21354)

134 (patient$ adj5 (attitude$ or belief$ or believ$ or experienc$ or opinion$ or perceiv$ or perception$ or perspective$ or preference$ or view or views or viewpoint$)).ti,ab. (253656)

135 or/132-134 (841986)

136 80 and 135 (3107)

137 131 or 136 (8322)

138 exp animals/ not humans/ (4609653)

139 (rat or rats or mouse or mice or rodent or rodents or swine or porcine or murine or sheep or lamb or lambs or ewe or ewes or pig or pigs or piglet or piglets or sow or sows or rabbit or rabbits or kitten or kittens or dog or dogs or puppy or puppies or monkey or monkeys or horse or horses or foal or foals or equine or calf or calves or cattle or heifer or heifers or hamster or hamsters or chicken or chickens or livestock or goat or goats).ti. (2075994)

140 138 or 139 (4948773)

141 137 not 140 (7989)

142 limit 141 to english language (7071)

Leggett H, *et al. BMJ Open* 2020; 10:e040378. doi: 10.1136/bmjopen-2020-040378
