# Supplementary material for: Quality of life following a lower limb reconstructive procedure: a protocol for the development of a conceptual framework
Source: BMJ Open. 2020 Dec 10;10(12):e040378. doi: 10.1136/bmjopen-2020-040378 (PMC7733194; doi:10.1136/bmjopen-2020-040378)
Supplement: Author's manuscript [file bmjopen-2020-040378.draft_revisions.pdf]

BMJ Open is committed to open peer review. As part of this commitment we make the peer review history of every article we publish publicly available.

When an article is published we post the peer reviewers' comments and the authors' responses online. We also post the versions of the paper that were used during peer review. These are the versions that the peer review comments apply to.

The versions of the paper that follow are the versions that were submitted during the peer review process. They are not the versions of record or the final published versions. They should not be cited or distributed as the published version of this manuscript.

BMJ Open is an open access journal and the full, final, typeset and author-corrected version of record of the manuscript is available on our site with no access controls, subscription charges or pay-per-view fees (http://bmjopen.bmj.com).

If you have any questions on BMJ Open's open peer review process please email info.bmjopen@bmj.com

BMJ Open

Quality of life following a lower limb reconstructive procedure: A protocol for the development of a conceptual framework

Journal:	*BMJ Open*
Manuscript ID	bmjopen-2020-040378
Article Type:	Protocol
Date Submitted by the Author:	13-May-2020
Complete List of Authors:	Leggett, Heather; University of York, York Trials Unit<br>Scantlebury, Arabella; University of York, Centre for Reviews & Dissemination<br>Sharma, Hemant; Hull Royal Infirmary, Trauma and Orhtopadics<br>Hewitt, Catherine; The University of York, Health Sciences<br>Harden, Melissa; University of York, Centre for reviews and dissemination<br>McDaid, Catriona; University of York, York Trials Unit
Keywords:	QUALITATIVE RESEARCH, Quality in health care < HEALTH SERVICES ADMINISTRATION & MANAGEMENT, Adult orthopaedics < ORTHOPAEDIC & TRAUMA SURGERY, Protocols & guidelines < HEALTH SERVICES ADMINISTRATION & MANAGEMENT

SCHOLARONE™
Manuscripts

I, the Submitting Author has the right to grant and does grant on behalf of all authors of the Work (as defined in the below author licence), an exclusive licence and/or a non-exclusive licence for contributions from authors who are: i) UK Crown employees; ii) where BMJ has agreed a CC-BY licence shall apply, and/or iii) in accordance with the terms applicable for US Federal Government officers or employees acting as part of their official duties; on a worldwide, perpetual, irrevocable, royalty-free basis to BMJ Publishing Group Ltd ("BMJ") its licensees and where the relevant Journal is co-owned by BMJ to the co-owners of the Journal, to publish the Work in this journal and any other BMJ products and to exploit all rights, as set out in our licence.

The Submitting Author accepts and understands that any supply made under these terms is made by BMJ to the Submitting Author unless you are acting as an employee on behalf of your employer or a postgraduate student of an affiliated institution which is paying any applicable article publishing charge ("APC") for Open Access articles. Where the Submitting Author wishes to make the Work available on an Open Access basis (and intends to pay the relevant APC), the terms of reuse of such Open Access shall be governed by a Creative Commons licence – details of these licences and which Creative Commons licence will apply to this Work are set out in our licence referred to above.

Other than as permitted in any relevant BMJ Author's Self Archiving Policies, I confirm this Work has not been accepted for publication elsewhere, is not being considered for publication elsewhere and does not duplicate material already published. I confirm all authors consent to publication of this Work and authorise the granting of this licence.

For peer review only

Quality of life following a lower limb reconstructive procedure: A protocol for the development of a conceptual framework

Leggett H[a*], Scantlebury A[a], Sharma H[b], Hewitt C[a], Harden M[c], McDaid C[a], the PROLLIT study collaborators.

a. York Trials Unit, Health Sciences, University of York, York, UK.

b. Department of Trauma & Orthopaedics, Hull Royal Infirmary, Hull, UK

c. Centre for Reviews and Dissemination, University of York, York, UK

** Corresponding author. heather.leggett@york.ac.uk*

Word count

3996

Abstract

Introduction

Lower limb conditions requiring reconstructive surgery can be either congenital or acquired from trauma, infection or other medical conditions. Patient reported outcome measures (PROMS) are often used by healthcare professionals to assess the impact of a patient's condition (and the treatment) on quality of life and health related quality of life. However, we are not aware of any measures developed specifically for people requiring lower limb reconstructive surgery. As such, it is not clear the extent to which current PROMs accurately and specifically measure the outcomes that are important to these patients.

Methods and analysis

This study will undertake a three phase approach: to explore what is important to patients with regards to quality of life (Phase 1), ascertain whether current measures adequately capture these experiences (Phase 2), and if not, begin the development of a new measure (Phase 3). The population of interest is people requiring, undergoing or after undergoing reconstructive surgery for a lower limb condition. Phase 1, described here, aims to develop a conceptual framework to identify and map what is important to this group with regards to social interactions, employment, perceived health and quality of life after condition onset/injury and throughout recovery. The conceptual framework will be developed through three steps: (Step A) a qualitative evidence synthesis, (Step B) a qualitative study with patients and staff to explore patient's views and experiences of lower limb reconstructive surgery and (Step C) a roundtable discussion with key stakeholders where findings from the qualitative evidence synthesis (Step A) and qualitative study (Step B) will be brought together and used to finalise the conceptual framework.

Ethics consideration and dissemination

Ethical approval will be sought for the qualitative data collection (Step B). Findings from the qualitative evidence synthesis and the qualitative data collection will be submitted for peer-reviewed publication in academic journals, and presented at relevant academic conferences.

For peer review only - http://bmjopen.bmj.com/site/about/guidelines.xhtml

PROSPERO registration number: CRD42019139587

ISRCTN Registration number: ISRCTN75201623

Article Summary

Strengths and limitations of this study

- The conceptual framework will be strengthened by combining findings from a qualitative evidence synthesis and qualitative interviews/focus groups.
- Patient and orthopaedic staff participants will be recruited from five Hospitals in England.
- Study findings will be used to establish the areas of importance for patients' quality of life following a lower limb reconstructive procedure and inform the development of future patient reported outcome measures if required.

For peer review only

For peer review only - http://bmjopen.bmj.com/site/about/guidelines.xhtml

Introduction

There are several different lower limb conditions requiring reconstructive surgery. These can be either congenital or acquired from trauma, infection or other medical conditions. Conditions may include, but are not limited to bone loss, joint contracture, nonunion, malunion or bone deformity. The experience of living with a lower limb condition as well as the impact of initial trauma or condition onset and any follow-on consequences may have negative impacts on an individual's' quality of life (QOL)[1.] Consequently, it is important to understand the experience of patients during and after reconstructive surgery for a lower limb condition in order to measure treatment effectiveness and to monitor and improve care, care management, resource management and health policy. Healthcare professionals and researchers regularly utilise patient-reported outcome measures (PROMs) with patients. They often include assessments of a patient's specific condition and the impact (of the condition and treatment) on both quality of life and health related quality of life [2,3]. These are important aspects for knowing if, and how to improve quality of care for patients[3].

Commonly used outcome measures for this population are related to musculoskeletal function such as the Olerud-Molander Ankle Score (OMAS[4]), or generic measures such as the Disability Rating Index (DRI[5]), Short Form 12 Health Survey (SF-12[6]), The Short Form 36 Health Survey (SF-36[7]), EQ-5D-5L[8] or the Sickness Impact Profile[9] (SIP). These tools have been effective in capturing and reporting the quality of life of patients[10,11]. More recently, the Patient-Reported Outcomes Measurement Information System (PROMIS) Physical Function measure was validated in patients with an orthopaedic trauma to a lower extremity[11,12]. However, this measure did not include quality of life assessment and was limited to patients with an isolated lower extremity fracture only (with either surgery or non-operative closed treatment). Research is currently also underway to develop a PROM specifically for children and adolescents with lower limb deformities[13] as well as for adult patients undergoing a lower limb circular frame fixation[14]. Whilst the work conducted by Antonios and colleagues[14] will be relevant to a subset of our population it excludes a large number of patients undergoing other reconstructive treatments. The focus of existing PROMS means it is not clear whether they accurately and specifically measure the outcomes that are important to patients requiring or undergoing reconstructive surgery for a lower limb condition.

Despite increased awareness surrounding the value of including patients in the development of PROMS, patients are still being underused[15] and there is concern over the extent to which existing PROMS used with patients who have experienced a lower limb condition are fit for the purpose of accurately capturing important patient experiences. We are not aware of any measures developed specifically for people with conditions requiring reconstructive surgery. This study, known as "PROLLIT" (Patient Reported Outcome Measure for Lower Limb Reconstruction) will address this evidence gap by adopting a three phase approach (Figure 1) to explore what is important to patients with regards to quality of life (Phase 1); ascertain whether current measures adequately capture these experiences (Phase 2); and if not, to begin the development of a new measure (Phase 3).

In this protocol paper we outline Phase 1 of the PROLLIT study, which will involve developing a conceptual framework related to patients requiring, undergoing or after undergoing reconstructive surgery for a lower limb condition (Figure 2).

For peer review only - http://bmjopen.bmj.com/site/about/guidelines.xhtml

Insert Figure 1

Insert Figure 2.

Methods. Development of a Conceptual Framework

In phase 1, our three stepped approach for developing a conceptual framework involves a qualitative evidence synthesis (Step A), a qualitative study with health professionals and patients (Step B) and a roundtable discussion with key stakeholders (Step c; Figure 2). The conceptual framework will be developed to identify and map what is important to patients requiring, undergoing or after undergoing reconstructive surgery for a lower limb condition with regards to social interactions, employment, perceived health and quality of life after injury and throughout recovery.

For readability, we describe the process of developing our conceptual framework in three steps. However, it is important to recognise that this is not a linear study and that framework development will be on-going and iterative with each 'step' of the study influencing one-another and contributing to the framework's overall development. For example, the findings of the qualitative evidence synthesis (Step A) will result in an initial framework. These findings will then be used to develop the topic guides for the qualitative study (Step B), which, through semi-structured interviews will draw upon the direct experience of health professionals and patients to explore in more depth, and where appropriate add to the findings of the qualitative evidence synthesis. In step C the findings of the qualitative evidence synthesis and qualitative study will then be combined and taken to a roundtable discussion with key stakeholders, the purpose of which will be to refine and finalise our conceptual framework (Figure 2).

The conceptual framework will then be used to inform the wider PROLLIT study, where we will, depending on whether patient priorities are being met by existing PROMS, develop a new PROM for people requiring, undergoing or who have undergone reconstructive surgery for a lower limb condition (Figure 1).

A patient and public involvement and engagement group will be engaged throughout all stages of the study. Patient and public involvement and engagement will include, but is not limited to, reviewing the documentation for the qualitative data collection as well as sense checking the domains of importance identified from the qualitative evidence synthesis and the qualitative data synthesis and the final conceptual framework. This will ensure that everything is appropriate and comprehensible from a patient's perspective.

For peer review only - http://bmjopen.bmj.com/site/about/guidelines.xhtml

Step A. Qualitative Evidence Synthesis

This qualitative evidence synthesis will explore what is important to patients with regards to quality of life after experiencing a lower limb reconstructive procedure. The Cochrane Handbook and guidance for Qualitative Evidence Synthesis (QES) published by the Cochrane Qualitative and Implementation Methods Group[16] provides the methodological framework for the design and implementation of this review. This QES will only include studies of 'good quality' based on the developed inclusion and exclusion criteria. The QES protocol complies with the requirements of the Preferred Reporting Items for Systematic Reviews and Meta-Analyses Protocol (PRISMA-P[17]) as far as possible as PRISMA focuses on reviews of interventions. The QES findings (and final report) will be reported according to ENTREQ guidelines[18], as appropriate for qualitative evidence syntheses.

Eligibility

Sample- Patients (Adults - 16+) requiring, undergoing or who have undergone reconstructive surgery for a lower limb condition (leg, ankle or foot). Conditions may include:

- Infection - A fracture fixation which becomes infected
- Nonunion - A fracture which does not heal
- Malunion / Deformity - A fracture which does not heal in correct position. Any acquired or congenital condition leading to bone deformity.
- Leg length discrepancy or bone loss
- Congenital lower limb deformities
- Joint contracture
- Lower limb injuries where further limb reconstruction is required.
- Poly-trauma patients (as long as one of the above criteria are met).

Time since condition onset/ reconstructive surgery: patients will be included at any time point after condition onset. For example, participants will be included if they are still in hospital, under hospital outpatient care regarding their lower limb condition/reconstructive surgery or if they have been discharged with no further follow-up.

Phenomenon of interest- Quality of life, including (but not limited to) social interactions, employment, perceived health and quality of life after condition onset/injury and throughout recovery.

Design- We will include qualitative studies which use established qualitative methods such as interviews or focus groups and use established qualitative analytical approaches (e.g. thematic analysis, framework analysis, grounded theory) to analyse the findings. We will include mixed-methods research which meets the criteria of a qualitative study outlined above. We will exclude opinion pieces, commentaries, case studies, guidelines, audits, observational studies, RCTs and other experimental designs.

Evaluation- We will include studies reporting on patient's attitudes, perspectives and behaviours surrounding quality of life in the broadest sense. This will encompass people's experiences of the condition (symptoms/pain/recovery), experiences of treatment, their physical, mental, emotional, social, daily and professional functioning (effect on working and any financial difficulties), as well as outcome expectations.

Research Type- Qualitative evidence synthesis.

For peer review only - http://bmjopen.bmj.com/site/about/guidelines.xhtml

For peer review only

Search strategy for the identification of relevant studies

The following databases will be searched: MEDLINE (Including: Epub Ahead of Print, In-Process & Other Non-Indexed Citations, MEDLINE Daily and MEDLINE, via Ovid), Embase (Ovid), PsycINFO (Ovid), and CINAHL Complete (Ebsco). A preliminary search strategy has been developed for Ovid MEDLINE in conjunction with an Information Specialist from the Centre for Reviews and Dissemination at the University of York (Supplementary file 1). The strategy in brief consists of a set of terms for our population of interest combined using the Boolean operator AND, with a set of terms covering the research methods of interest (qualitative and mixed methods). The search terms relating to qualitative methods are based upon previous search strategies evaluated by Shaw et al[19]. This strategy will be adapted for the remaining databases. Searches will be run from database inception until present, and will be limited to publications in English. A manual search of the reference list of included studies will be undertaken to identify other studies meeting the inclusion criteria.

Selection of studies and data extraction

All results will be imported into EndNote reference management software (Clarivate Analytics (formerly Thomson Reuters), Philadelphia, PA, USA) and de-duplicated. Titles of records will be screened by a single researcher (task divided between three researchers) to remove obviously irrelevant records. Using the stated eligibility criteria, the abstract of each record included from the title screen stage will be independently reviewed by two researchers (task divided between three researchers). The full text of each record included from the abstract screen stage will then be independently screened by two researchers, against the eligibility criteria (task divided between three researchers). Discrepancies will be resolved by discussion with a third researcher.

Data extraction will be undertaken on the included studies using Excel for key study details. A data extraction template will be developed and piloted within the research team in order to summarise key information and persistent themes within studies. Key information will include author names, design or methods including method of data collection and analysis, participant characteristics and results. Results extracted will relate to patients' condition, treatments, outcomes, experiences, attitudes and thoughts surrounding quality of life and other relevant outcomes using the NVIVO software. Data extraction will be undertaken by one researcher and checked by a second.

Quality assessment

Guidance by the Cochrane Qualitative and Implementation Methods Group recommends researchers assess methodological strengths and weaknesses[20]. In line with this guidance, the Critical Appraisal Skills Programme[21] checklist will be used to assess the included studies. Each article will be assessed independently by two researchers. Any difficulties in identifying each study's methodological quality will be resolved by discussing the paper in question with a third researcher. GRADE-CERQual[22] will be used to assess the strength of the review's findings.

Data analysis and synthesis

Data synthesis will be in line with the Cochrane Qualitative and Implementation Methods Group guidelines for qualitative evidence synthesis[23]. Key study characteristics including details of the population, setting, methods and study quality will be tabulated and described narratively. Thematic synthesis will be undertaken to analyse the qualitative data; data analysis will be facilitated by the

For peer review only - http://bmjopen.bmj.com/site/about/guidelines.xhtml

use of NVIVO. Thematic synthesis lends itself well to this research question and the type of data we anticipate finding since it allows for the synthesis and integration of both thin and more richly detailed data into descriptive and analytic themes[25]. The thematic synthesis will be based on the entirety of the 'results' section of each included study and will involve a three stage process[24]. Firstly, both verbatim comments (quotes) and author observations (author interpretations) from included studies will be coded. Secondly, the identification of common findings from individual studies will lead to the creation of researcher developed descriptive themes to describe these findings. The first two stages will be undertaken independently by three researchers and then discussed as a group. Thirdly, analytical themes will be developed to describe and interpret these themes, moving beyond the content of the original articles. For example, multiple instances of patient reported frustration, fatigue, depression and anxiety may be grouped together as a theme entitled 'Psychological Impact' and then further explored to understand the impact of this on QOL. This is an iterative process achieved through independent and group discussions of the implications of each theme.

After our initial thematic analysis, we will hold a roundtable discussion amongst the research team to develop a framework where we will identify the important areas related to quality of life for patients requiring, undergoing or after undergoing reconstructive surgery for a lower limb condition that were identified in the synthesis. These findings will be used to inform topic guides for the qualitative data collection in Step B and will contribute to the development of the initial, hypothesised conceptual framework in step C (see Figure 2).

Step B. Qualitative Data Collection

Design

A qualitative study using semi-structured interviews and focus groups will be undertaken with patients and staff (orthopaedic clinicians, specialist nurses and physiotherapists) working with orthopaedic patients at five NHS hospitals in England. It is anticipated that including the perspectives of both staff and patients, from a range of NHS hospitals will provide a more encompassing and detailed exploration of important QOL related outcomes for patients.

Participants and recruitment (selection of participants)

A sampling frame will be developed through collaboration with lead clinicians from the participating sites. Patients will be identified and invited to participate by orthopaedic clinicians, specialist nurses, physiotherapists or research nurses at five participating NHS hospitals in England. The five sites are orthopaedic departments of varying size, with different orthopaedic specialities and are situated throughout England to offer geographical spread. A purposive sampling strategy will be adopted to ensure maximum variation according to: age, gender, length of time since injury or condition onset, different lower limb conditions, trauma/condition severity and treatment received. Care will be taken to ensure that those who are requiring, undergoing or who have undergone reconstructive surgery for a lower limb condition, and will have received care through the NHS at one of the participating sites are included. To achieve maximum variation we aim to recruit between 50-75 patient participants across the five sites (10-15 at each site) and 10 staff members (2 from each site), however analysis will stop or continue beyond this if saturation has/ has not been reached. Data collection will continue until saturation is reached and no new information is gained in the interviews/focus groups with regards to factors and outcomes surrounding patients' quality of life.

For peer review only - http://bmjopen.bmj.com/site/about/guidelines.xhtml

Staff (orthopaedic clinicians, specialist nurses and physiotherapists) will be invited to participate in the study by either direct contact from the researcher if they meet the clinician when visiting participating sites for patient interviews or through the lead contact at each site who will be identified through the PROLLIT study's PI's professional contacts.

Procedure

Patients will be invited to take part in either a one-to-one interview with a researcher (face-to-face or via telephone) or a focus group with a researcher and a maximum of seven other participants. Individual semi-structured interviews or focus groups (approx. 4-8 people) will be undertaken with the patient participants and will be led by an experienced qualitative researcher. The interviews/focus groups will be arranged at a time most appropriate for the participants. A mix of focus groups and interviews for patients allows for flexibility in recruitment and scheduling the interviewers/focus groups. The group setting of the focus group may also provide an opportunity for patients to feel more relaxed and open, as well as enabling patients to bounce thoughts and discussion points off each other. Staff will be invited to take part in a one-to-one interview with the researcher either face-to-face or via telephone. Due to the limited time availability of working staff it will not be practical to arrange focus groups.

It is anticipated that interviews will last between 30 and 60 minutes and focus groups between 1 to 2 hours. Topic guides will be used to guide the interview/focus groups. These will be developed from a literature review of the area, our QES findings and the initial hypothesised conceptual framework developed from step A. The topic guides will be reviewed by experts in the field and our patient, public involvement and engagement group. The topic guide will use open-ended questions to explore key health-related quality of life factors and other factors relevant to patients who have experienced a lower limb condition that requires or required reconstructive surgery.

Through the use of the topic guide and questioning, concept elicitation will be undertaken in the interview/focus group by asking the participants questions which explore their thoughts, attitudes and beliefs surrounding what is important to them with regards to quality of life in relation to requiring, undergoing or after reconstructive surgery for a lower limb condition. Important factors may include physical, social and psychological well-being as well as job and lifestyle related factors. The staff will be asked to discuss, from their perspective, what they perceive to be important treatment outcomes and goals for patients.

Ethics and consent

We will ensure that participants have read the information sheet provided and have a full understanding of the study before consent is obtained. Participants will be invited to take part and will be reminded of their right to withdraw at any time. All participants (staff and patients) will be informed that participation is voluntary and that their involvement and responses will remain anonymous. Informed consent will be taken prior to each interview or focus group. Verbal consent will be obtained on an on-going basis during interviews/focus groups Participants will be reminded that the interviews/focus groups will be audio-recorded, quotations may be used and published, but that all identifiable information will be removed. All participants will be provided with a unique ID to maintain their anonymity.

For peer review only - http://bmjopen.bmj.com/site/about/guidelines.xhtml

Analysis

Data will be analysed at the University of York by qualitative researchers (AS, HL), who will adopt an iterative approach to data analysis, involving regular discussion between the research team. The qualitative data will be recorded and transcribed verbatim with data analysis facilitated by the use of NVIVO. Quotations will be used where appropriate to provide evidence for the conclusions drawn when reporting the study's findings.

We have chosen to analyse our data using Framework analysis[26] a method which allows for flexibility during the analysis process, as data can either be collected and then analysed, or analysed alongside data collection. The analysis will involve data being sifted, charted and sorted in accordance with key issues and themes. In this study, the focus will be on drawing out and grouping into key themes the important factors and outcomes relating to patients' self-reported quality of life, as well as staff views on important treatment and outcome goals for patients. This will enable the research team to understand in greater depth, and from a psychological standpoint, the areas that are important to patients. To achieve this, our analysis will follow the five stages of framework analysis as outlined by Richie and Spencer[25]: 1. familiarisation; 2. identifying a thematic framework; 3. indexing; 4. charting; and 5. mapping and interpretation. In stage 1 the researchers will become familiarised with and immersed in the transcripts by reading them through multiple times. Following this, coding of the transcripts will begin by identifying emerging themes related to patient's quality of life. Coding will be deductively based on a-priori themes from the QES findings where appropriate, whilst also inductively allowing for new themes to emerge which may influence the conceptual framework development. These will be influenced by and be complimentary to the findings from the QES. Multiple iterations of this step will enable the development of a thematic framework (stage 2). Indexing and charting (stage 3, 4) will begin whereby portions of the text that relate to a particular theme will be identified and charted according to the theme it corresponds to. Finally, in stage 5 mapping and interpretation involves analysis of the characteristics of each charted theme through graphical representation of the themes and how they relate to each other[26]. At the end of our analysis, the findings of the framework will be combined with those of the qualitative evidence synthesis (step B) and used to develop the conceptual framework (step C).

Step C. Conceptual Framework Development

An initial conceptual framework will be developed using findings from the qualitative evidence synthesis and qualitative study. As previously discussed, findings will then be combined by the research team and a 'hypothesised conceptual framework' will be taken to a roundtable discussion with key stakeholders. Key stakeholders will include members of the project advisory panel, patient and public involvement and engagement group and research team.

Methodological guidance whilst developing the conceptual framework will be sought from guidelines and conceptual framework method papers as required[27-30]. The knowledge and experience of the advisory panel and the patient and public involvement and engagement group will also be vital for the final refinement of the conceptual framework in step C and they will be invited to work closely alongside the research team at this stage. The final conceptual framework will represent what is important to patients with regards to social interactions, employment, perceived health and quality of life after injury and throughout recovery after lower limb reconstructive surgery.

For peer review only - http://bmjopen.bmj.com/site/about/guidelines.xhtml

Advisory Panel and Patient and Public Involvement and Engagement

An Advisory Panel has been convened, consisting of orthopaedic surgeons, physiotherapists and methodologists to provide support, opinion and insight at key times during the project and to support recruitment to the study. The group will be contacted through a variety of methods such as emails, face-to-face meetings, telephone and video conferencing. An advisory patient and public involvement and engagement group will also be engaged throughout all stages of the study. Patient and public involvement and engagement will include, but is not limited to, reviewing the documentation for the qualitative data collection as well as sense checking the domains of importance identified from the qualitative evidence synthesis and the qualitative data synthesis and the final conceptual framework. This will ensure that everything is appropriate and comprehensible from a patient's perspective. Regular meetings within the research team will also be held to discuss study development and address any issues.

Ethics and dissemination

Only step B requires ethical approval which has been given favourable opinion from South Central – Berkshire Research Ethics committee (REF:20/SC/0114). All necessary local research governance approvals will be obtained at each of the five sites prior to data collection. The interviews/focus groups will not impact on the care of the patients or the staff professionally. All participants will be provided with written information about the study and informed consent will be obtained. Step A is registered on the international prospective register of systematic reviews (PROSPERO) database (CRD number: CRD42019139587) and step B is registered on the International Standard Randomised Controlled Trial Number Registry (ISRCTN75201623). We intend to publish the QES findings and the findings from the qualitative study in a peer reviewed journal, disseminate at relevant conferences and provide feedback on the findings to participants who are interested and other patient groups and organisations.

Acknowledgements

The authors would like to thank the advisory panel and the advisory patient and public involvement and engagement group who provided insight into defining the population of interest, refining the study design and providing feedback on key materials for step B.

Sponsorship

The study is being sponsored by The University of York

Indemnity

The University of York will provide legal liability cover for their employed staff and participants in the study. Non negligent harm will not be covered.

Funding

This project is funded through Hull University Teaching Hospitals NHS Trust.

Competing interests

Dr. McDaid reports grants from Hull University Teaching Hospitals NHS Trust, during the conduct of the study; grants from British Orthopaedic Association, outside the submitted work.

For peer review only

Dr. Sharma reports grants from Health & Technology Assessment, during the conduct of the study; grants from BBraun, personal fees from Biocomposites and personal fees from Orthofix, outside the submitted work.

Ms Harden, Dr Leggett, Dr Scantlebury and Professor Hewitt have nothing to disclose.

Contributors

CM, CH, HS were involved in the study conception. CM, HS, CH, HL and AS designed the study. MH developed the search strategy for the QES. HL drafted the manuscript with input from AS, CM, HS, MH and CH. The PROLLITT Collaborators provided methodological and clinical advice. All authors and the PROLLIT study collaborators reviewed and approved the final manuscript.

PROLLIT Study Collaborators

Joy Adamson, PhD (The University of York), Georgina Jones, PhD (Leeds Beckett University), Kim Cocks, PhD (KCStats Consultancy), Joel Gagnier, PhD (University of Michigan), Paul Harwood, MBChB (Leeds Teaching Hospitals NHS Trust), David Ferguson, MBChB (South Tees Hospitals, NHS Trust), Reggie Hamdy, MBChB (McGill University) and Nando Ferriera, PhD (Stellenbosch University).

For peer review only

References

1. Giannoudis PV, Harwood PJ, Kontakis G, et al. Long-term quality of life in trauma patients following the full spectrum of tibial injury (fasciotomy, closed fracture, grade IIIB/IIIC open fracture and amputation). *Injury* 2009;**40**:213-19.
2. Black N. Patient reported outcome measures could help transform healthcare. *BMJ* 2013;**346**:f167.
3. Brédart A, Marrel A, Abetz-Webb L, et al. Interviewing to develop Patient-Reported Outcome (PRO) measures for clinical research: eliciting patients' experience. *Health Qual Life Outcomes* 2014;**12**:15.
4. Olerud C, Molander H. A scoring scale for symptom evaluation after ankle fracture. *Arch Orthop Trauma Surg* 1984;**103**:190-94.
5. Salen BA, Nordemar R. The Disability Rating Index: an instrument for the assessment of disability in clinical settings. *J Clin Epidemiol* 1994;**47**:1423-35.
6. Ware Jr JE, Kosinski M, Keller SD. A 12-Item Short-Form Health Survey: construction of scales and preliminary tests of reliability and validity. *Med Care* 1996:220-233.
7. Ware JE. Scoring the SF-36. *SF-36 Health Survey: Manual and Interpretation Guide* 1993.
8. Herdman M, Gudex C, Lloyd A, et al. Development and preliminary testing of the new five-level version of EQ-5D (EQ-5D-5L). *Qual Life Res* 2011;**20**:1727-36.
9. Bergner M, Bobbitt RA, Kressel S, et al. The sickness impact profile: conceptual formulation and methodology for the development of a health status measure. *Int J Health Serv* 1976;**6**:393-415.
10. Hoogendoorn J, van der Werken C. Grade III open tibial fractures: functional outcome and quality of life in amputees versus patients with successful reconstruction. *Injury* 2001;**32**:329-34.
11. MacKenzie EJ, Bosse MJ, Pollak AN, et al. Long-term persistence of disability following severe lower-limb trauma: Results of a seven-year follow-up. *JBJS* 2005;**87**:1801-09.
12. Rothrock NE, Kaat AJ, Vrahas MS, et al. Validation of PROMIS physical function instruments in patients with an orthopaedic trauma to a lower extremity. *J Orthop Trauma* 2019;**33**:377-83.
13. Chhina H, Klassen A, Kopec JA, et al. International multiphase mixed methods study protocol to develop a patient-reported outcome instrument for children and adolescents with lower limb deformities. *BMJ open* 2019;**9**:e027079.
14. Antonios T, Barker A, Ibrahim I, et al. A Systematic Review of Patient-reported Outcome Measures Used in Circular Frame Fixation. *Strategies Trauma Limb Reconstr* 2019;**14**:34.
15. Wiering B, de Boer D, Delnoij D. Patient involvement in the development of patient-reported outcome measures: a scoping review. *Health Expect* 2017;**20**:11-23.
16. Harris JL, Booth A, Cargo M, et al. Cochrane Qualitative and Implementation Methods Group guidance series—paper 2: methods for question formulation, searching, and protocol development for qualitative evidence synthesis. *J Clin Epidemiol* 2018;**97**:39-48.
17. Moher D, Shamseer L, Clarke M, et al. Preferred reporting items for systematic review and meta-analysis protocols (PRISMA-P) 2015 statement. *Syst Rev* 2015;**4**:1.
18. Tong A, Flemming K, McInnes E, et al. Enhancing transparency in reporting the synthesis of qualitative research: ENTREQ. *BMC Med Res Methodol* 2012;**12**:181.
19. Shaw RL, Booth A, Sutton AJ, et al. Finding qualitative research: an evaluation of search strategies. *BMC Med Res Methodol* 2004;**4**:5.
20. Noyes J, Booth A, Cargo M, et al. Cochrane Qualitative and Implementation Methods Group guidance series—paper 1: introduction. *J Clin Epidemiol* 2018;**97**:35-38.
21. Checklist CQ. Oxford, UK: Critical Appraisal Skills Programme (CASP), 2019.
22. Lewin S, Bohren M, Rashidian A, et al. Applying the GRADE-CERQual approach: making an overall CERQual assessment of confidence and creating a Summary of Qualitative Findings table. *Implement Sci* 2018;**13**(S1):10.

For peer review only - http://bmjopen.bmj.com/site/about/guidelines.xhtml

23. Harden A, Thomas J, Cargo M, et al. Cochrane Qualitative and Implementation Methods Group guidance series—paper 5: methods for integrating qualitative and implementation evidence within intervention effectiveness reviews. *J Clin Epidemiol* 2018;**97**:70-78.
24. Thomas J, Harden A. Methods for the thematic synthesis of qualitative research in systematic reviews. *BMC Med Res Methodol* 2008;**8**:45.
25. Flemming K, Booth A, Garside R, et al. Qualitative evidence synthesis for complex interventions and guideline development: clarification of the purpose, designs and relevant methods. *BMJ Glob Health* 2019;**4**(S1): e000882
26. Ritchie J. Spencer, l.(1994). Qualitative data analysis for applied policy research. *Bryman and Burgess (Eds), Analysing Qualitative Data* 1994:173-94.
27. Food, Administration D. Guidance for industry: patient-reported outcome measures: use in medical product development to support labeling claims. *Fed Regist* 2009;**74**:65132-33.
28 Peters M, Potter CM, Kelly L, et al. The long-term conditions questionnaire: conceptual framework and item development. *Patient Relat Outcome Meas* 2016;**7**:109.
29. Patrick DL, Burke LB, Gwaltney CJ, et al. Content validity—establishing and reporting the evidence in newly developed patient-reported outcomes (PRO) instruments for medical product evaluation: ISPOR PRO good research practices task force report: part 1—eliciting concepts for a new PRO instrument. *Value Health* 2011;**14:**967-77.
30. Alam R, Figueiredo SM, Balvardi S, et al. Development of a patient-reported outcome measure of recovery after abdominal surgery: a hypothesized conceptual framework. *Surg Endosc* 2018;**32**:4874-85.

For peer review only

For peer review only - http://bmjopen.bmj.com/site/about/guidelines.xhtml

Figure legends:

Figure 1: The PROLLIT study

Figure 2: The three phases of developing a conceptual framework for patients requiring, undergoing or after undergoing reconstructive surgery for a lower limb condition that are outlined in this paper

For peer review only

For peer review only - http://bmjopen.bmj.com/site/about/guidelines.xhtml

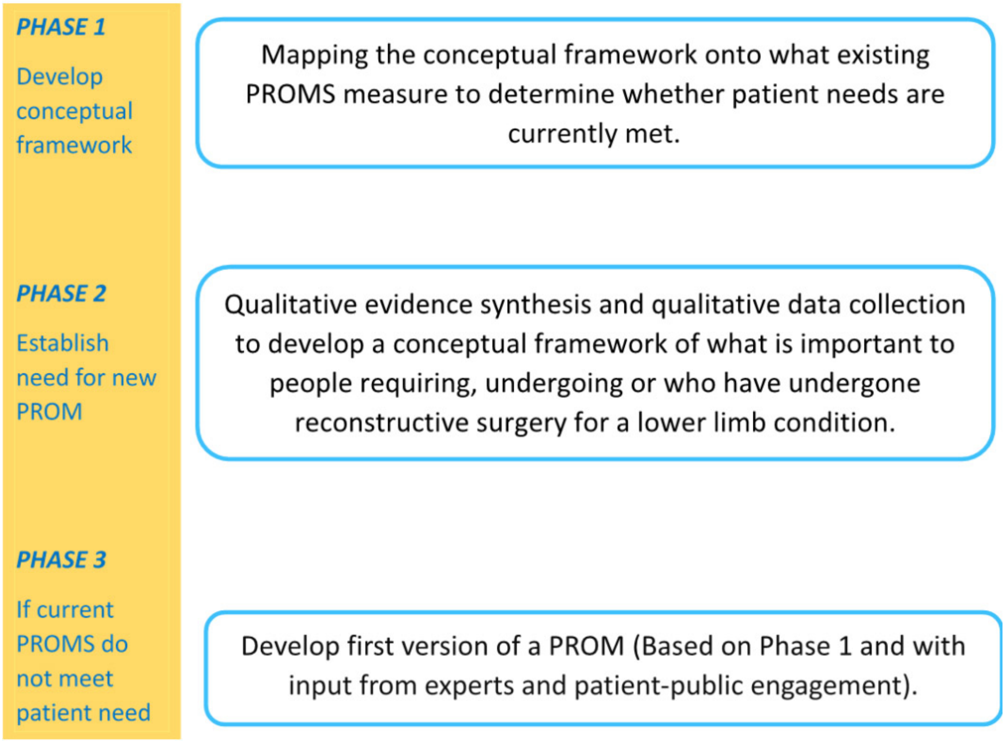

Figure 1. The PROLLIT study

90x90mm (300 x 300 DPI)

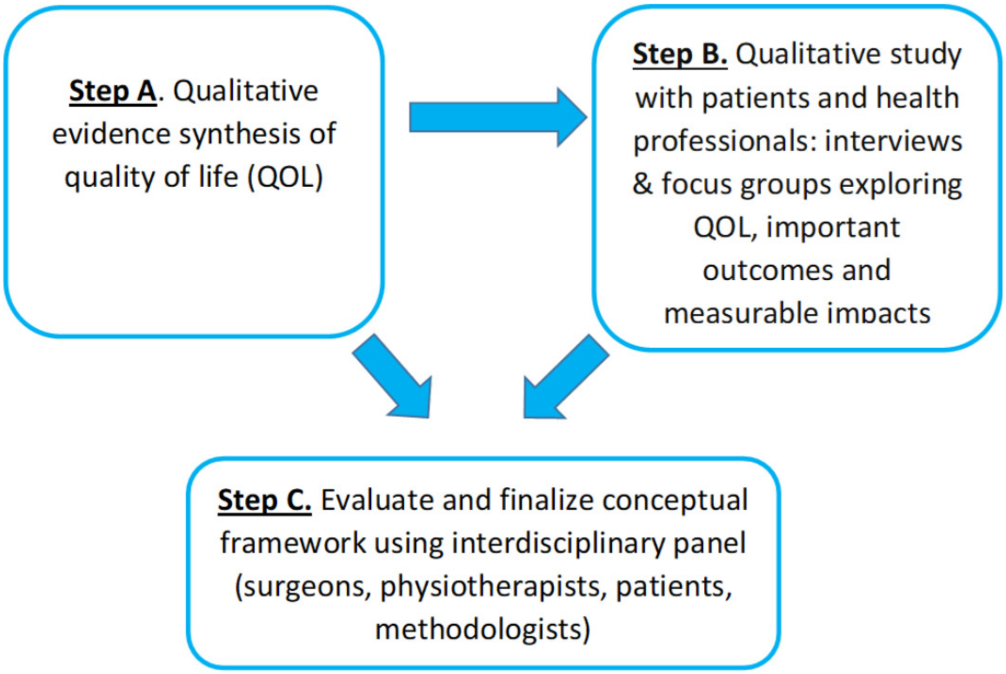

Figure 2. The three phases of developing a conceptual framework for patients requiring, undergoing or after undergoing reconstructive surgery for a lower limb condition that are outlined in this paper

90x90mm (300 x 300 DPI)

Database: Ovid MEDLINE(R) ALL <1946 to August 16, 2019>

19/08/2019

DRAFT Search Strategy:

----

1 Ankle Fractures/ (1253)

2 Femoral Fractures/ (15922)

3 Tibial Fractures/ (14766)

4 1 or 2 or 3 (29498)

5 ((fractur$ or trauma$) adj3 (lower extremit$ or lower limb$)).ti,ab. (2992)

6 ((fractur$ or trauma$) adj3 (leg or legs or thigh$ or knee or knees or shin or shins or shinbone$ or foot or midfoot or hindfoot or feet or ankle or ankles or anklebone$ or pilon or heel or heels or heelbone$ or toe or toes)).ti,ab. (9355)

7 ((fractur$ or trauma$) adj3 (femur$ or femoral or tibia$ or fibula$ or patella$ or kneecap$ or knee cap$)).ti,ab. (33008)

8 ((fractur$ or trauma$) adj3 (metatarsal$ or metatarsus or metatarsi or tarsus or tarsi or tarsal or tarsals or cuneiform or cuboid or navicular or calcane$ or talus or astragalus or tali or bimalleol$ or malleol$ or trimalleol$)).ti,ab. (5249)

9 ((Pott$ or Cotton$) adj fractur$).ti,ab. (30)

10 or/5-9 (46979)

11 lower extremity/ or exp foot/ or knee/ or leg/ or thigh/ (146797)

12 leg injuries/ or ankle injuries/ or foot injuries/ or knee injuries/ (38409)

13 exp Leg Bones/ (92594)

14 exp Foot Bones/ (17063)

15 11 or 12 or 13 or 14 (273200)

16 (lower extremit$ or lower limb$).ti,ab. (94764)

17 (leg or legs or thigh$ or knee or knees or shin or shins or shinbone$ or foot or midfoot or hindfoot or feet or ankle or ankles or anklebone$ or pilon or heel or heels or heelbone$ or toe or toes).ti,ab. (396803)

18 (femur$ or femoral or tibia$ or fibula$ or patella$ or kneecap$ or knee cap$).ti,ab. (237589)

19 (metatarsal$ or metatarsus or metatarsi or tarsus or tarsi or tarsal or tarsals or cuneiform or cuboid or navicular or calcane$ or talus or astragalus or tali or bimalleol$ or malleol$ or trimalleol$).ti,ab. (36596)

20 or/15-19 (717215)

21 Fractures, Bone/ (62396)

22 20 and 21 (18698)

23 (fractur$ or trauma$).ti,ab. (546587)

24 15 and 23 (39512)

For peer review only

25 exp Fracture Dislocation/ (2087)
26 Fractures, Avulsion/ (117)
27 Fractures, Closed/ (3275)
28 Fractures, Comminuted/ (2413)
29 Fractures, Compression/ (2080)
30 Fractures, Malunited/ (1551)
31 Fractures, Multiple/ (114)
32 Fractures, Open/ (5278)
33 Fractures, Stress/ (3216)
34 exp Fractures, Ununited/ (10494)
35 Intra-Articular Fractures/ (1104)
36 Periprosthetic Fractures/ (941)
37 Fracture Healing/ (12723)
38 Crush Injuries/ (121)
39 (crush$ adj2 (injur$ or trauma$ or fractur$)).ti,ab. (3676)
40 or/25-39 (43086)
41 20 and 40 (18153)
42 exp Multiple Trauma/ (12521)
43 (polytrauma$ or poly-trauma$ or multitrauma$ or multi-trauma$).ti,ab. (4627)
44 ((complex$ or complicat$ or severe$ or severity or serious or major or multiple) adj2 (fractur$ or trauma$)).ti,ab. (36622)
45 or/42-44 (48056)
46 20 and 45 (7375)
47 4 or 10 or 22 or 24 or 41 or 46 (87320)
48 ((bone$ or fracture$) adj3 (heal or heals or healed or healing)).ti,ab. (21408)
49 ((bone$ or fracture$) adj3 (union$ or nonunion$ or non union$ or ununite$)).ti,ab. (8182)
50 ((bone$ or fracture$) adj3 (malunion$ or mal union$ or deform$)).ti,ab. (4430)
51 (osteomyelitis or ((bone$ or fracture$) adj3 infect$)).ti,ab. (28503)
52 exp Osteomyelitis/ (22216)
53 or/48-52 (66572)
54 20 and 53 (21088)
55 ((femur$ or femoral or tibia$ or fibula$ or patella$ or kneecap$ or knee cap$) adj3 (heal or heals or healed or healing)).ti,ab. (1389)
56 ((metatarsal$ or metatarsus or metatarsi or tarsus or tarsi or tarsal or tarsals or cuneiform or cuboid or navicular or calcane$ or talus or astragalus or tali or bimalleol$ or malleol$ or trimalleol$) adj3 (heal or heals or healed or healing)).ti,ab. (107)

For peer review only

For peer review only - http://bmjopen.bmj.com/site/about/guidelines.xhtml

57 ((femur$ or femoral or tibia$ or fibula$ or patella$ or kneecap$ or knee cap$) adj3 (union$ or nonunion$ or non union$ or ununite$)).ti,ab. (1699)

58 ((metatarsal$ or metatarsus or metatarsi or tarsus or tarsi or tarsal or tarsals or cuneiform or cuboid or navicular or calcane$ or talus or astragalus or tali or bimalleol$ or malleol$ or trimalleol$) adj3 (union$ or nonunion$ or non union$ or ununite$)).ti,ab. (151)

59 ((femur$ or femoral or tibia$ or fibula$ or patella$ or kneecap$ or knee cap$) adj3 (malunion$ or mal union$ or deform$)).ti,ab. (1822)

60 ((metatarsal$ or metatarsus or metatarsi or tarsus or tarsi or tarsal or tarsals or cuneiform or cuboid or navicular or calcane$ or talus or astragalus or tali or bimalleol$ or malleol$ or trimalleol$) adj3 (malunion$ or mal union$ or deform$)).ti,ab. (528)

61 ((femur$ or femoral or tibia$ or fibula$ or patella$ or kneecap$ or knee cap$) adj3 (osteomyelitis or infect$)).ti,ab. (1622)

62 ((metatarsal$ or metatarsus or metatarsi or tarsus or tarsi or tarsal or tarsals or cuneiform or cuboid or navicular or calcane$ or talus or astragalus or tali or bimalleol$ or malleol$ or trimalleol$) adj3 (osteomyelitis or infect$)).ti,ab. (360)

63 or/55-62 (7267)

64 Limb Salvage/ (3799)

65 ((limb or limbs) adj3 (restor$ or reconstruct$ or salvag$)).ti,ab. (8371)

66 Ilizarov Technique/ (1280)

67 External Fixators/ (5652)

68 Orthopedic Fixation Devices/ (4991)

69 Fracture Fixation/ (18000)

70 Bone Lengthening/ (2175)

71 Ilizarov.ti,ab. (2107)

72 (external adj2 (fixat$ or frame$ or cage$)).ti,ab. (9626)

73 (circular adj2 (fixat$ or frame$ or cage$)).ti,ab. (609)

74 Taylor Spatial Frame$.ti,ab. (194)

75 TSF.ti,ab. (1209)

76 True Lok Hex.ti,ab. (0)

77 TLHex.ti,ab. (0)

78 or/64-77 (43999)

79 20 and 78 (20689)

80 47 or 54 or 63 or 79 (108579)

81 exp qualitative research/ (47998)

82 "Surveys and Questionnaires"/ (433413)

83 Self Report/ (27877)

84 exp Attitude/ (546190)

For peer review only - http://bmjopen.bmj.com/site/about/guidelines.xhtml

85 Focus Groups/ (27342)

86 Ethnology/ (1581)

87 discourse analysis.mp. (1718)

88 content analysis.mp. (25061)

89 ethnographic research.mp. (887)

90 ethnological research.mp. (7)

91 constant comparative method.mp. (1463)

92 qualitative validity.mp. (19)

93 purposive sample.mp. (3194)

94 observational method$.mp. (713)

95 field stud$.mp. (14522)

96 theoretical sampl$.mp. (635)

97 phenomenology.mp. (8689)

98 phenomenological research.mp. (431)

99 life experience$.mp. (5101)

100 or/81-99 (959292)

101 interview$.mp. or interviews/ or Interviews as Topic/ (365560)

102 qualitative.mp. (224736)

103 or/101-102 (512625)

104 100 or 103 (1295594)

105 ethnograph$.mp. (10148)

106 phenomenol$.mp. (24867)

107 grounded theory.mp. (10833)

108 (grounded adj (theor$ or study or studies or research or analys?s)).mp. (10995)

109 (emic or etic or hermeneutic$ or heuristic$ or semiotic$).mp. (16188)

110 (data adj1 saturat$).tw. (1054)

111 participant observ$.tw. (4226)

112 (action research or cooperative inquir$ or co operative inquir$ or co-operative inquir$).mp. (3997)

113 (field adj (study or studies or research or observation$)).tw. (18880)

114 theoretical sampl$.mp. (635)

115 (purpos$ adj4 sampl$).mp. (13410)

116 (focus adj group$).mp. (47371)

117 (account or accounts or unstructured or open-ended or open ended or text$ or narrative$).mp. (603046)

118 (life world or life-world or conversation analys?s or personal experience$ or theoretical saturation).mp. (14630)

For peer review only

119 lived experience$.tw. (5075)

120 (theme$ or thematic).mp. (97515)

121 (observational adj (method$ or research or stud$)).mp. (142328)

122 questionnaire$.mp. (691305)

123 content analysis.mp. (25061)

124 thematic analysis.mp. (15722)

125 discourse analys?s.mp. (1756)

126 ((discourse$ or discurs$) adj3 analys?s).tw. (2062)

127 (constant adj (comparative or comparison)).mp. (4283)

128 narrative analys?s.mp. (1157)

129 or/105-128 (1573717)

130 104 or 129 (2205208)

131 80 and 130 (5967)

132 survey$.ti,ab. (598311)

133 (mixed method$ or multimethod$ or multi-method$ or multi method$).mp. (21354)

134 (patient$ adj5 (attitude$ or belief$ or believ$ or experienc$ or opinion$ or perceiv$ or perception$ or perspective$ or preference$ or view or views or viewpoint$)).ti,ab. (253656)

135 or/132-134 (841986)

136 80 and 135 (3107)

137 131 or 136 (8322)

138 exp animals/ not humans/ (4609653)

139 (rat or rats or mouse or mice or rodent or rodents or swine or porcine or murine or sheep or lamb or lambs or ewe or ewes or pig or pigs or piglet or piglets or sow or sows or rabbit or rabbits or kitten or kittens or dog or dogs or puppy or puppies or monkey or monkeys or horse or horses or foal or foals or equine or calf or calves or cattle or heifer or heifers or hamster or hamsters or chicken or chickens or livestock or goat or goats).ti. (2075994)

140 138 or 139 (4948773)

141 137 not 140 (7989)

142 limit 141 to english language (7071)

For peer review only - http://bmjopen.bmj.com/site/about/guidelines.xhtml

PRISMA-P 2015 Checklist

This checklist has been adapted for use with protocol submissions to *Systematic Reviews* from Table 3 in Moher D et al: Preferred reporting items for systematic review and meta-analysis protocols (PRISMA-P) 2015 statement. *Systematic Reviews* 2015 **4**:1

Section/topic	#	Checklist item	Information reported Yes	Information reported No	Line number(s)
**ADMINISTRATIVE INFORMATION**					
**Title**					
Identification	1a	Identify the report as a protocol of a systematic review	☒	☐	P4. L19-20
Update	1b	If the protocol is for an update of a previous systematic review, identify as such	☐	☒	
**Registration**	2	If registered, provide the name of the registry (e.g., PROSPERO) and registration number in the Abstract	☒	☐	P2 L11
**Authors**					
Contact	3a	Provide name, institutional affiliation, and e-mail address of all protocol authors; provide physical mailing address of corresponding author	☒	☐	P1. L11-20
Contributions	3b	Describe contributions of protocol authors and identify the guarantor of the review	☒	☐	P11. L13-19
**Amendments**	4	If the protocol represents an amendment of a previously completed or published protocol, identify as such and list changes; otherwise, state plan for documenting important protocol amendments	☐	☒	
**Support**					
Sources	5a	Indicate sources of financial or other support for the review	☒	☐	P10. L53
Sponsor	5b	Provide name for the review funder and/or sponsor	☒	☐	P10. L45. P10. L53
Role of sponsor/funder	5c	Describe roles of funder(s), sponsor(s), and/or institution(s), if any, in developing the protocol	☐	☒	
**INTRODUCTION**					
**Rationale**	6	Describe the rationale for the review in the context of what is already known	☒	☐	P3. L5-54
**Objectives**	7	Provide an explicit statement of the question(s) the review will address with reference to participants, interventions, comparators, and outcomes (PICO)	☒	☐	P5. L5-6
**METHODS**					

BioMed Central
The Open Access Publisher

For peer review only - http://bmjopen.bmj.com/site/about/guidelines.xhtml

Section/topic	#	Checklist item	Information reported: Yes	Information reported: No	Line number(s)
**Eligibility criteria**	8	Specify the study characteristics (e.g., PICO, study design, setting, time frame) and report characteristics (e.g., years considered, language, publication status) to be used as criteria for eligibility for the review	☒	☐	P5. L20-60
**Information sources**	9	Describe all intended information sources (e.g., electronic databases, contact with study authors, trial registers, or other grey literature sources) with planned dates of coverage	☒	☐	P6. L 5-17
**Search strategy**	10	Present draft of search strategy to be used for at least one electronic database, including planned limits, such that it could be repeated	☒	☐	Supplementary file
STUDY RECORDS					
Data management	11a	Describe the mechanism(s) that will be used to manage records and data throughout the review	☒	☐	P6. L21
Selection process	11b	State the process that will be used for selecting studies (e.g., two independent reviewers) through each phase of the review (i.e., screening, eligibility, and inclusion in meta-analysis)	☒	☐	P6. L22-30
Data collection process	11c	Describe planned method of extracting data from reports (e.g., piloting forms, done independently, in duplicate), any processes for obtaining and confirming data from investigators	☒	☐	P6. L31-39
**Data items**	12	List and define all variables for which data will be sought (e.g., PICO items, funding sources), any pre-planned data assumptions and simplifications	☒	☐	P6. L36-38
**Outcomes and prioritization**	13	List and define all outcomes for which data will be sought, including prioritization of main and additional outcomes, with rationale	☒	☐	P6. L31-38
**Risk of bias in individual studies**	14	Describe anticipated methods for assessing risk of bias of individual studies, including whether this will be done at the outcome or study level, or both; state how this information will be used in data synthesis	☒	☐	P6. L44-50
DATA					
**Synthesis**	15a	Describe criteria under which study data will be quantitatively synthesized	☐	☒	
	15b	If data are appropriate for quantitative synthesis, describe planned summary measures, methods of handling data, and methods of combining data from studies, including any planned exploration of consistency (e.g., I^2, Kendall's tau)	☐	☒	
	15c	Describe any proposed additional analyses (e.g., sensitivity or subgroup analyses, meta-regression)	☐	☒	
	15d	If quantitative synthesis is not appropriate, describe the type of summary planned	☒	☐	P6/7. L55-26
**Meta-bias(es)**	16	Specify any planned assessment of meta-bias(es) (e.g., publication bias across studies, selective reporting within studies)	☐	☒	
**Confidence in cumulative evidence**	17	Describe how the strength of the body of evidence will be assessed (e.g., GRADE)	☒	☐	P6.L50

For peer review only

BioMed Central The Open Access Publisher

BMJ Open

Quality of life following a lower limb reconstructive procedure: A protocol for the development of a conceptual framework

Journal:	*BMJ Open*
Manuscript ID	bmjopen-2020-040378.R1
Article Type:	Protocol
Date Submitted by the Author:	05-Oct-2020
Complete List of Authors:	Leggett, Heather; University of York, York Trials Unit<br>Scantlebury, Arabella; University of York, Centre for Reviews & Dissemination<br>Sharma, Hemant; Hull Royal Infirmary, Trauma and Orhtopadics<br>Hewitt, Catherine; The University of York, Health Sciences<br>Harden, Melissa; University of York, Centre for reviews and dissemination<br>McDaid, Catriona; University of York, York Trials Unit
**Primary Subject Heading**:	Qualitative research
Secondary Subject Heading:	Research methods
Keywords:	QUALITATIVE RESEARCH, Quality in health care < HEALTH SERVICES ADMINISTRATION & MANAGEMENT, Adult orthopaedics < ORTHOPAEDIC & TRAUMA SURGERY, Protocols & guidelines < HEALTH SERVICES ADMINISTRATION & MANAGEMENT

SCHOLARONE™
Manuscripts

I, the Submitting Author has the right to grant and does grant on behalf of all authors of the Work (as defined in the below author licence), an exclusive licence and/or a non-exclusive licence for contributions from authors who are: i) UK Crown employees; ii) where BMJ has agreed a CC-BY licence shall apply, and/or iii) in accordance with the terms applicable for US Federal Government officers or employees acting as part of their official duties; on a worldwide, perpetual, irrevocable, royalty-free basis to BMJ Publishing Group Ltd ("BMJ") its licensees and where the relevant Journal is co-owned by BMJ to the co-owners of the Journal, to publish the Work in this journal and any other BMJ products and to exploit all rights, as set out in our licence.

The Submitting Author accepts and understands that any supply made under these terms is made by BMJ to the Submitting Author unless you are acting as an employee on behalf of your employer or a postgraduate student of an affiliated institution which is paying any applicable article publishing charge ("APC") for Open Access articles. Where the Submitting Author wishes to make the Work available on an Open Access basis (and intends to pay the relevant APC), the terms of reuse of such Open Access shall be governed by a Creative Commons licence – details of these licences and which Creative Commons licence will apply to this Work are set out in our licence referred to above.

Other than as permitted in any relevant BMJ Author's Self Archiving Policies, I confirm this Work has not been accepted for publication elsewhere, is not being considered for publication elsewhere and does not duplicate material already published. I confirm all authors consent to publication of this Work and authorise the granting of this licence.

For peer review only

Quality of life following a lower limb reconstructive procedure: A protocol for the development of a conceptual framework

Leggett H[a*], Scantlebury A[a], Sharma H[b], Hewitt C[a], Harden M[c], McDaid C[a], the PROLLIT study collaborators.

a. York Trials Unit, Health Sciences, University of York, York, UK.

b. Department of Trauma & Orthopaedics, Hull University Teaching Hospitals, Hull, UK

c. Centre for Reviews and Dissemination, University of York, York, UK

** Corresponding author. heather.leggett@york.ac.uk*

Word count

4018

Abstract

Introduction

Lower limb conditions requiring reconstructive surgery can be either congenital or acquired from trauma, infection or other medical conditions. Patient reported outcome measures (PROMS) are often used by healthcare professionals to assess the impact of a patient's condition (and the treatment) on quality of life. However, we are not aware of any measures developed specifically for people requiring lower limb reconstructive surgery. As such, it is not clear the extent to which current PROMs accurately and specifically measure the outcomes that are important to these patients.

Methods and analysis

The 'PROLLIT' (Patient Reported Outcome Measure for Lower Limb Reconstruction) involves three phases: to explore what is important to patients with regards to quality of life (phase 1), ascertain whether current measures adequately capture these experiences (phase 2) and if not begin the development of a new patient reported outcome measure (phase 3). The population of interest is people requiring, undergoing or after undergoing reconstructive surgery for a lower limb condition. In this paper we describe Phase 1, which, aims to develop a conceptual framework to identify and map what is important to this group with regards to social interactions, employment, perceived health and quality of life after condition onset/injury and throughout recovery. The conceptual framework will be developed through three steps: (Step A) a qualitative evidence synthesis, (Step B) a qualitative study with patients and staff to explore patient's views and experiences of lower limb reconstructive surgery and (Step C) a roundtable discussion with key stakeholders where findings from the qualitative evidence synthesis (Step A) and qualitative study (Step B) will be brought together and used to finalise the conceptual framework.

For peer review only - http://bmjopen.bmj.com/site/about/guidelines.xhtml

Ethics consideration and dissemination

Ethical approval has been sought for the qualitative data collection (Step B). Findings from the qualitative evidence synthesis and the qualitative data collection will be submitted for peer-reviewed publication in academic journals, and presented at relevant academic conferences.

PROSPERO registration number: CRD42019139587

ISRCTN Registration number: ISRCTN75201623

Article Summary

Strengths and limitations of this study

- A conceptual framework will be developed to establish the areas of importance for patients' quality of life and will determine if there is a need to develop a new patient reported outcome measure.
- A multi-methods approach will be used to develop the conceptual framework, increasing the robustness of the study.
- Both patient and staff opinions on important areas relating to quality of life after a lower limb reconstruction will be sought which will offer a more encompassing and detailed exploration.
- Due to resource constraints only English language studies will be eligible for inclusion in the review, introducing the risk of language bias.
- There is a risk that some individuals may be more likely than others to agree to take part in the qualitative study limiting generalisability, though strategies are in place to mitigate against this.

For peer review only

For peer review only - http://bmjopen.bmj.com/site/about/guidelines.xhtml

Introduction

There are several different lower limb conditions requiring reconstructive surgery. These can be congenital or acquired from trauma, infection or other medical conditions. Conditions may include, but are not limited to bone loss, joint contracture, nonunion, malunion or bone deformity. Limb reconstruction is the orthopaedic subspecialty, treating sequel of complex trauma and congenital conditions; deformity correction, nonunion, malunion, bone infections, limb lengthening and complex trauma. Individuals with a lower limb condition often experience mobility impairments as well as pain, discomfort, sleep disturbance, anxiety and low mood which can affect their daily life and reduce their employment and social engagement opportunities[1] **.** Therefore, it is not surprising that the experience of living with a lower limb condition as well as the impact of initial trauma or condition onset and any follow-on consequences may have negative impacts on an individual's quality of life (QOL)[2.] Consequently, it is important to understand the experience of patients during and after reconstructive surgery for a lower limb condition in order to measure treatment effectiveness and to monitor and improve care, care management, resource management and health policy. Healthcare professionals and researchers regularly utilise patient-reported outcome measures (PROMs) with patients. They often include assessments of a patient's specific condition and the impact (of the condition and treatment) on quality of life [3 4]. These are important aspects for knowing if, and how to improve quality of care for patients[4].

Commonly used outcome measures for this population are related to musculoskeletal function such as the Olerud-Molander Ankle Score (OMAS[5]), or generic measures such as the Disability Rating Index (DRI[6]), Short Form 12 Health Survey (SF-12[7]), The Short Form 36 Health Survey (SF-36[8]), EQ-5D-5L[9] or the Sickness Impact Profile[10] (SIP). These tools have been used in capturing and reporting the quality of life of patients who have experienced lower limb reconstruction[11 12]. More recently, the Patient-Reported Outcomes Measurement Information System (PROMIS) Physical Function measure was validated in patients with an orthopaedic trauma to a lower extremity[12 13]. However, this measure did not include quality of life assessment and was limited to patients with an isolated lower extremity fracture only (with either surgery or non-operative closed treatment). Research is currently also underway to develop a PROM specifically for children and adolescents with lower limb deformities[14] as well as for adult patients undergoing a lower limb circular frame fixation[15]. Whilst the work conducted by Antonios and colleagues[15] will be relevant to a subset of our population it excludes a large number of patients undergoing other reconstructive treatments. The focus of existing PROMS means it is not clear whether they accurately and specifically measure the outcomes that are important to patients requiring or undergoing reconstructive surgery for a lower limb condition. As such, the need for a measure specifically designed to capture outcomes important to those requiring or undergoing reconstructive surgery for a lower limb condition may be warranted and needs further exploration.

Despite increased awareness surrounding the value of including patients in the development of PROMS, patients are not being fully engaged[16] and there is concern over the extent to which existing PROMS used with patients who have experienced a lower limb condition are fit for the purpose of accurately capturing important patient experiences. We are not aware of any measures developed specifically for people with conditions requiring reconstructive surgery. This study, known as "PROLLIT" (Patient Reported Outcome Measure for Lower Limb Reconstruction) will address this evidence gap by adopting a three phase approach (Figure 1) to explore what is important to patients with regards to quality of life (Phase 1); ascertain whether current measures adequately capture these experiences (Phase 2); and if not, to begin the development of a new measure (Phase 3).

For peer review only - http://bmjopen.bmj.com/site/about/guidelines.xhtml

In this protocol paper we outline Phase 1 of the PROLLIT study, which will involve developing a conceptual framework related to patients requiring, undergoing or after undergoing reconstructive surgery for a lower limb condition (Figure 2).

Insert Figure 1

Insert Figure 2.

For peer review only

Methods. Development of a Conceptual Framework

In phase 1, our three stepped approach for developing a conceptual framework involves a qualitative evidence synthesis (Step A), a qualitative study with health professionals and patients (Step B) and a roundtable discussion with key stakeholders (Step C; Figure 2). The conceptual framework will be developed to identify and map what is important to patients requiring, undergoing or after undergoing reconstructive surgery for a lower limb condition with regards to social interactions, employment, perceived health and quality of life after injury and throughout recovery.

For readability, we describe the process of developing our conceptual framework in three steps. However, it is important to recognise that this is not a linear study and that framework development will be on-going and iterative with each ‘step’ of the study influencing the other and contributing to the framework’s overall development. For example, the findings of the qualitative evidence synthesis (Step A) will result in an initial framework. These findings will then be used to develop the topic guides for the qualitative study (Step B), which, through semi-structured interviews will draw upon the direct experience of health professionals and patients to explore in more depth, and where appropriate add to the findings of the qualitative evidence synthesis. In step C the findings of the qualitative evidence synthesis and qualitative study will then be combined and taken to a roundtable discussion with key stakeholders, the purpose of which will be to refine and finalise our conceptual framework (Figure 2).

The conceptual framework will then be used to inform the wider PROLLIT study, where we will, depending on whether patient priorities are being met by existing PROMS, develop a new PROM for people requiring, undergoing or who have undergone reconstructive surgery for a lower limb condition (Figure 1).

Step A. Qualitative Evidence Synthesis

This qualitative evidence synthesis will explore what is important to patients with regards to quality of life after experiencing a lower limb reconstructive procedure. The Cochrane Handbook and guidance for Qualitative Evidence Synthesis (QES) published by the Cochrane Qualitative and Implementation Methods Group[17] provides the methodological framework for the design and implementation of this review. This QES will only include studies of ‘good quality’ based on the

developed inclusion and exclusion criteria. The QES protocol complies with the requirements of the Preferred Reporting Items for Systematic Reviews and Meta-Analyses Protocol (PRISMA-P[18]) as far as possible as PRISMA focuses on reviews of interventions. The QES findings (and final report) will be reported according to ENTREQ guidelines[19], as appropriate for qualitative evidence syntheses.

Eligibility

Sample- Patients (Adults - 16+) requiring, undergoing or who have undergone limb reconstructive surgery for a lower limb condition (leg, ankle or foot). Conditions may include:

- Infection - A fracture fixation which becomes infected
- Nonunion - A fracture which does not heal
- Malunion / Deformity - A fracture which does not heal in correct position. Any acquired or congenital condition leading to bone deformity.
- Leg length discrepancy or bone loss
- Congenital lower limb deformities
- Joint contracture
- Lower limb injuries where further limb reconstruction is required.
- Poly-trauma patients (as long as one of the above criteria are met).

- Patients who have undergone a lower limb amputation will not be included

Time since condition onset/ reconstructive surgery: patients will be included at any time point after condition onset. For example, participants will be included if they are still in hospital, under hospital outpatient care regarding their lower limb condition/reconstructive surgery or if they have been discharged with no further follow-up.

Phenomenon of interest- Quality of life, including (but not limited to) social interactions, employment, perceived health and quality of life after condition onset/injury and throughout recovery.

Design- We will include qualitative studies which use established qualitative methods such as interviews or focus groups and use established qualitative analytical approaches (e.g. thematic analysis, framework analysis, grounded theory) to analyse the findings. We will include mixed-methods research which meets the criteria of a qualitative study outlined above. We will exclude opinion pieces, commentaries, case studies, guidelines, audits, observational studies, RCTs and other experimental designs.

Evaluation- We will include studies reporting on patient's attitudes, perspectives and behaviours surrounding quality of life in the broadest sense. This will encompass people's experiences of the condition (symptoms/pain/recovery), experiences of treatment, their physical, mental, emotional, social, daily and professional functioning (effect on working and any financial difficulties), as well as outcome expectations.

Research Type- Qualitative evidence synthesis.

For peer review only - http://bmjopen.bmj.com/site/about/guidelines.xhtml

Search strategy for the identification of relevant studies

The following databases will be searched: MEDLINE (Including: Epub Ahead of Print, In-Process & Other Non-Indexed Citations, MEDLINE Daily and MEDLINE, via Ovid), Embase (Ovid), PsycINFO (Ovid), and CINAHL Complete (Ebsco). A preliminary search strategy has been developed for Ovid MEDLINE in conjunction with an Information Specialist from the Centre for Reviews and Dissemination at the University of York (Supplementary file 1). The strategy in brief consists of a set of terms for our population of interest combined using the Boolean operator AND, with a set of terms covering the research methods of interest (qualitative and mixed methods). The search terms relating to qualitative methods are based upon previous search strategies evaluated by Shaw et al[20]. This strategy will be adapted for the remaining databases. Searches will be run from database inception until present, and will be limited to publications in English. A manual search of the reference list of included studies will be undertaken to identify other studies meeting the inclusion criteria.

Selection of studies and data extraction

All results will be imported into EndNote reference management software (Clarivate Analytics (formerly Thomson Reuters), Philadelphia, PA, USA) and de-duplicated. Titles of records will be screened by a single researcher (task divided between three researchers) to remove obviously irrelevant records. Using the stated eligibility criteria, the abstract of each record included from the title screen stage will be independently reviewed by two researchers (task divided between three researchers). The full text of each record included from the abstract screen stage will then be independently screened by two researchers, against the eligibility criteria (task divided between three researchers). Discrepancies will be resolved by discussion with a third researcher.

Data extraction will be undertaken on the included studies using Excel for key study details. A data extraction template will be developed and piloted within the research team in order to summarise key information and persistent themes within studies. Key information will include author names, design or methods including method of data collection and analysis, participant characteristics and results. Results extracted will relate to patients' condition, treatments, outcomes, experiences, attitudes and thoughts surrounding quality of life and other relevant outcomes using the NVIVO software. Data extraction will be undertaken by one researcher and checked by a second.

Quality assessment

Guidance by the Cochrane Qualitative and Implementation Methods Group recommends researchers assess methodological strengths and weaknesses[21]. In line with this guidance, the Critical Appraisal Skills Programme[22] checklist will be used to assess the included studies. Each article will be assessed independently by two researchers. Any difficulties in identifying each study's methodological quality will be resolved by discussing the paper in question with a third researcher. GRADE-CERQual[23] will be used to assess the strength of the review's findings.

Data analysis and synthesis

Data synthesis will be in line with the Cochrane Qualitative and Implementation Methods Group guidelines for qualitative evidence synthesis[24]. Key study characteristics including details of the population, setting, methods and study quality will be tabulated and described narratively. Thematic synthesis will be undertaken to analyse the qualitative data; data analysis will be facilitated by the

For peer review only - http://bmjopen.bmj.com/site/about/guidelines.xhtml

use of NVIVO. Thematic synthesis lends itself well to this research question and the type of data we anticipate finding since it allows for the synthesis and integration of both thin and more richly detailed data into descriptive and analytic themes[25]. The thematic synthesis will be based on the entirety of the 'results' section of each included study and will involve a three stage process[26]. Firstly, both verbatim comments (quotes) and author observations (author interpretations) from included studies will be coded. Secondly, the identification of common findings from individual studies will lead to the creation of researcher developed descriptive themes to describe these findings. The first two stages will be undertaken independently by three researchers and then discussed as a group. Thirdly, analytical themes will be developed to describe and interpret these themes, moving beyond the content of the original articles. For example, multiple instances of patient reported frustration, fatigue, depression and anxiety may be grouped together as a theme entitled 'Psychological Impact' and then further explored to understand the impact of this on QOL. This is an iterative process achieved through independent and group discussions of the implications of each theme.

After our initial thematic analysis, we will hold a roundtable discussion amongst the research team to develop a framework where we will identify the important areas related to quality of life for patients requiring, undergoing or after undergoing reconstructive surgery for a lower limb condition that were identified in the synthesis. These findings will be used to inform topic guides for the qualitative data collection in Step B and will contribute to the development of the initial, hypothesised conceptual framework in step C (see Figure 2).

Step B. Qualitative Data Collection

Design

A qualitative study using semi-structured interviews and focus groups will be undertaken with patients and staff (orthopaedic clinicians, specialist nurses and physiotherapists) working with orthopaedic patients at five NHS hospitals in England. It is anticipated that including the perspectives of both staff and patients, from a range of NHS hospitals will provide a more encompassing and detailed exploration of important QOL related outcomes for patients.

Participants and recruitment (selection of participants)

A sampling frame will be developed through collaboration with lead clinicians from the participating sites. Patients will be those who meet the 'Sample' eligibility criteria outlined in Step A above. Patients will be identified and invited to participate by orthopaedic clinicians, specialist nurses, physiotherapists or research nurses. The five sites are orthopaedic departments of varying size, with different orthopaedic specialities and are situated throughout England to offer geographical spread. A purposive sampling strategy will be adopted to ensure maximum variation according to: age, gender, length of time since injury or condition onset, different lower limb conditions, trauma/condition severity and treatment received. Care will be taken to ensure that those who are requiring, undergoing or who have undergone reconstructive surgery for a lower limb condition, and have received care through the NHS at one of the participating sites are included. To achieve maximum variation we aim to recruit between 50-75 patient participants across the five sites (10-15 at each site) and 10 staff members (2 from each site), however analysis will stop or continue beyond this if saturation has/ has not been reached. Data collection will continue until saturation is reached

For peer review only - http://bmjopen.bmj.com/site/about/guidelines.xhtml

and no new information is gained in the interviews/focus groups with regards to factors and outcomes surrounding patients' quality of life.

Staff (orthopaedic clinicians, specialist nurses and physiotherapists) will be invited to participate in the study by either direct contact from the researcher or through the lead contact at each site who will be identified through the PROLLIT study's PI's professional contacts. The anticipated start and end dates for data collection are November 2020-February 2021.

Procedure

Patients will be invited to take part in either a one-to-one interview with a researcher (virtual or via telephone) or a focus group with a researcher and a maximum of seven other participants. Individual semi-structured interviews or focus groups (approx. 4-8 people) will be undertaken with the patient participants and will be led by an experienced qualitative researcher. The interviews/focus groups will be arranged at a time most appropriate for the participants. A mix of focus groups and interviews for patients allows for flexibility in recruitment and scheduling the interviewers/focus groups. The group setting of the focus group may also provide an opportunity for patients to feel more relaxed and open, as well as enabling patients to bounce thoughts and discussion points off each other. Staff will be invited to take part in a one-to-one interview with the researcher either virtual or via telephone. Due to the limited time availability of working staff it will not be practical to arrange focus groups.

It is anticipated that interviews will last between 30 and 60 minutes and focus groups between 1 to 2 hours. Topic guides will be used to guide the interview/focus groups. These will be developed from a literature review of the area, our QES findings and the initial hypothesised conceptual framework developed from step A. The topic guides will be reviewed by experts in the field and our patient, public involvement and engagement group. The topic guide will use open-ended questions to explore key health-related quality of life factors and other factors relevant to patients who have experienced a lower limb condition that requires or required reconstructive surgery.

Through the use of the topic guide and questioning, concept elicitation will be undertaken in the interview/focus group by asking the participants questions which explore their thoughts, attitudes and beliefs surrounding what is important to them with regards to quality of life in relation to requiring, undergoing or after reconstructive surgery for a lower limb condition. Important factors may include physical, social and psychological well-being as well as job and lifestyle related factors. The staff will be asked to discuss, from their perspective, what they perceive to be important treatment outcomes and goals for patients.

Ethics and consent

We will ensure that participants have read the information sheet provided and have a full understanding of the study before consent is obtained. Participants will be invited to take part and will be reminded of their right to withdraw at any time. All participants (staff and patients) will be informed that participation is voluntary and that their involvement and responses will remain anonymous. Informed consent will be taken prior to each interview or focus group. Verbal consent will be obtained on an on-going basis during interviews/focus groups Participants will be reminded that the interviews/focus groups will be audio-recorded, quotations may be used and published, but that all identifiable information will be removed. All participants will be provided with a unique ID to maintain their anonymity.

For peer review only - http://bmjopen.bmj.com/site/about/guidelines.xhtml

Analysis

Data will be analysed at the University of York by qualitative researchers (AS, HL), who will adopt an iterative approach to data analysis, involving regular discussion between the research team. The qualitative data will be recorded and transcribed verbatim with data analysis facilitated by the use of NVIVO. Quotations will be used where appropriate to provide evidence for the conclusions drawn when reporting the study's findings.

We have chosen to analyse our data using Framework analysis[27] a method which allows for flexibility during the analysis process, as data can either be collected and then analysed, or analysed alongside data collection. The analysis will involve data being sifted, charted and sorted in accordance with key issues and themes. In this study, the focus will be on drawing out and grouping into key themes the important factors and outcomes relating to patients' self-reported quality of life, as well as staff views on important treatment and outcome goals for patients. This will enable the research team to understand in greater depth, and from a psychological standpoint, the areas that are important to patients. To achieve this, our analysis will follow the five stages of framework analysis as outlined by Richie and Spencer[25]: 1. familiarisation; 2. identifying a thematic framework; 3. indexing; 4. charting; and 5. mapping and interpretation. In stage 1 the researchers will become familiarised with and immersed in the transcripts by reading them through multiple times. Following this, coding of the transcripts will begin by identifying emerging themes related to patient's quality of life. Coding will be deductively based on a-priori themes from the QES findings where appropriate, whilst also inductively allowing for new themes to emerge which may influence the conceptual framework development. These will be influenced by and be complimentary to the findings from the QES. Multiple iterations of this step will enable the development of a thematic framework (stage 2). Indexing and charting (stage 3, 4) will begin whereby portions of the text that relate to a particular theme will be identified and charted according to the theme it corresponds to. Finally, in stage 5 mapping and interpretation involves analysis of the characteristics of each charted theme through graphical representation of the themes and how they relate to each other[27]. At the end of our analysis, the findings of the framework will be combined with those of the qualitative evidence synthesis (step B) and used to develop the conceptual framework (step C).

Step C. Conceptual Framework Development

An initial conceptual framework will be developed using findings from the qualitative evidence synthesis and qualitative study. As previously discussed, findings will then be combined by the research team and a 'hypothesised conceptual framework' will be taken to a roundtable discussion with key stakeholders. Key stakeholders will include members of the project advisory panel, patient and public involvement and engagement group and research team.

Methodological guidance whilst developing the conceptual framework will be sought from guidelines and conceptual framework method papers as required[28-31]. The knowledge and experience of the advisory panel and the patient and public involvement and engagement group will also be vital for the final refinement of the conceptual framework in step C and they will be invited to work closely alongside the research team at this stage. The final conceptual framework will represent what is important to patients with regards to social interactions, employment, perceived health and quality of life after injury and throughout recovery after lower limb reconstructive surgery.

For peer review only - http://bmjopen.bmj.com/site/about/guidelines.xhtml

For peer review only

Advisory Panel and Patient and Public Involvement and Engagement

An Advisory Panel has been convened, consisting of orthopaedic surgeons, physiotherapists and methodologists to provide support, opinion and insight at key times during the project and to support recruitment to the study. The group will be contacted through a variety of methods such as emails, face-to-face meetings, telephone and video conferencing. A patient and public involvement and engagement group will be also be engaged throughout all stages of the study. To maximise the diversity of the group we will aim to recruit people from a range of relevant patient support and clinical groups such as physiotherapy groups at each of the study sites. Patient and public involvement and engagement will include, but is not limited to, reviewing documentation for the qualitative study as well as sense checking the domains of importance identified from the qualitative evidence synthesis and the final conceptual framework. This will ensure that everything is appropriate and comprehensible from a patient's perspective.

Ethics and Dissemination

Only step B requires ethical approval which has been given favourable opinion from South Central – Berkshire Research Ethics committee (REF:20/SC/0114). All necessary local research governance approvals will be obtained at each of the five study sites prior to data collection. The interviews/focus groups will not impact on the care of the patients or the staff professionally. All participants will be provided with written information about the study and informed consent will be obtained. Step A is registered on the international prospective register of systematic reviews (PROSPERO) database (CRD number: CRD42019139587) and step B is registered on the International Standard Randomised Controlled Trial Number Registry (ISRCTN75201623). We intend to publish the QES findings and the findings from the qualitative study in a peer reviewed journal, disseminate at relevant conferences and provide feedback on the findings to participants who are interested and other patient groups and organisations.

Risks and Mitigations

Potential risks to the undertaking of this research pertain mainly to the recruitment of participants for Step B. Due to Covid 19 it may be harder to recruit patients due to reduced contact between hospital staff and patients which may make it difficult to achieve our desired sample size or to obtain a representative sample of patients. To mitigate this the researchers will closely monitor recruitment and if necessary ask those identifying patients to actively recruit certain types of patients. To mitigate the risk of Covid 19 to participants and researchers all interviews and focus groups will now be conducted either virtually via an online platform or via the telephone.

Acknowledgements

The authors would like to thank the advisory panel and the advisory patient and public involvement and engagement group who provided insight into defining the population of interest, refining the study design and providing feedback on key materials for step B.

Sponsorship

The study is being sponsored by The University of York

For peer review only - http://bmjopen.bmj.com/site/about/guidelines.xhtml

Indemnity

The University of York will provide legal liability cover for their employed staff and participants in the study. Non negligent harm will not be covered.

Funding

This project is funded through Research Capability funding from Hull University Teaching Hospitals NHS Trust.

Competing interests

Dr. McDaid reports grants from Hull University Teaching Hospitals NHS Trust, during the conduct of the study; grants from British Orthopaedic Association, outside the submitted work.

Dr. Sharma reports grants from Health & Technology Assessment, during the conduct of the study; grants from BBraun, personal fees from Biocomposites and personal fees from Orthofix, outside the submitted work.

Ms Harden, Dr Leggett, Dr Scantlebury and Professor Hewitt have nothing to disclose.

Contributors

CM, CH, HS were involved in the study conception. CM, HS, CH, HL and AS designed the study. MH developed the search strategy for the QES. HL drafted the manuscript with input from AS, CM, HS, MH and CH. The PROLLITT Collaborators provided methodological and clinical advice. All authors and the PROLLIT study collaborators reviewed and approved the final manuscript.

PROLLIT Study Collaborators

Joy Adamson, PhD (The University of York), Georgina Jones, PhD (Leeds Beckett University), Kim Cocks, PhD (KCStats Consultancy), Joel Gagnier, PhD (University of Michigan), Paul Harwood, MBChB (Leeds Teaching Hospitals NHS Trust), David Ferguson, MBChB (South Tees Hospitals, NHS Trust), Reggie Hamdy, MBChB (McGill University) and Nando Ferriera, PhD (Stellenbosch University).

For peer review only

References

1. Rees S, Tutton E, Achten J, et al. Patient experience of long-term recovery after open fracture of the lower limb: a qualitative study using interviews in a community setting. *BMJ open* 2019;9(10):e031261.
2. Giannoudis PV, Harwood PJ, Kontakis G, et al. Long-term quality of life in trauma patients following the full spectrum of tibial injury (fasciotomy, closed fracture, grade IIIB/IIIC open fracture and amputation). *Injury* 2009;40(2):213-19.
3. Black N. Patient reported outcome measures could help transform healthcare. *Bmj* 2013;346:f167.
4. Brédart A, Marrel A, Abetz-Webb L, et al. Interviewing to develop Patient-Reported Outcome (PRO) measures for clinical research: eliciting patients' experience. *Health and quality of life outcomes* 2014;12(1):15.
5. Olerud C, Molander H. A scoring scale for symptom evaluation after ankle fracture. *Archives of orthopaedic and traumatic surgery* 1984;103(3):190-94.
6. Salen BA, Nordemar R. The Disability Rating Index: an instrument for the assessment of disability in clinical settings. *Journal of clinical epidemiology* 1994;47(12):1423-35.
7. Ware Jr JE, Kosinski M, Keller SD. A 12-Item Short-Form Health Survey: construction of scales and preliminary tests of reliability and validity. *Medical care* 1996:220-33.
8. Ware JE. Scoring the SF-36. *SF-36 Health Survey: Manual and Interpretation Guide* 1993
9. Herdman M, Gudex C, Lloyd A, et al. Development and preliminary testing of the new five-level version of EQ-5D (EQ-5D-5L). *Quality of life research* 2011;20(10):1727-36.
10. Bergner M, Bobbitt RA, Kressel S, et al. The sickness impact profile: conceptual formulation and methodology for the development of a health status measure. *International Journal of Health Services* 1976;6(3):393-415.
11. Hoogendoorn J, van der Werken C. Grade III open tibial fractures: functional outcome and quality of life in amputees versus patients with successful reconstruction. *Injury* 2001;32(4):329-34.
12. MacKenzie EJ, Bosse MJ, Pollak AN, et al. Long-term persistence of disability following severe lower-limb trauma: Results of a seven-year follow-up. *JBJS* 2005;87(8):1801-09.
13. Rothrock NE, Kaat AJ, Vrahas MS, et al. Validation of PROMIS physical function instruments in patients with an orthopaedic trauma to a lower extremity. *Journal of orthopaedic trauma* 2019;33(8):377-83.
14. Chhina H, Klassen A, Kopec JA, et al. International multiphase mixed methods study protocol to develop a patient-reported outcome instrument for children and adolescents with lower limb deformities. *BMJ open* 2019;9(5):e027079.
15. Antonios T, Barker A, Ibrahim I, et al. A Systematic Review of Patient-reported Outcome Measures Used in Circular Frame Fixation. *Strategies in Trauma and Limb Reconstruction* 2019;14(1):34.
16. Wiering B, de Boer D, Delnoij D. Patient involvement in the development of patient-reported outcome measures: a scoping review. *Health Expectations* 2017;20(1):11-23.
17. Harris JL, Booth A, Cargo M, et al. Cochrane Qualitative and Implementation Methods Group guidance series—paper 2: methods for question formulation, searching, and protocol development for qualitative evidence synthesis. *Journal of clinical epidemiology* 2018;97:39-48.
18. Moher D, Shamseer L, Clarke M, et al. Preferred reporting items for systematic review and meta-analysis protocols (PRISMA-P) 2015 statement. *Systematic reviews* 2015;4(1):1.
19. Tong A, Flemming K, McInnes E, et al. Enhancing transparency in reporting the synthesis of qualitative research: ENTREQ. *BMC medical research methodology* 2012;12(1):181.
20. Shaw RL, Booth A, Sutton AJ, et al. Finding qualitative research: an evaluation of search strategies. *BMC Medical Research Methodology* 2004;4(1):5. doi: 10.1186/1471-2288-4-5
21. Noyes J, Booth A, Cargo M, et al. Cochrane Qualitative and Implementation Methods Group guidance series—paper 1: introduction. *Journal of clinical epidemiology* 2018;97:35-38.
22. Checklist CQ. Oxford, UK: Critical Appraisal Skills Programme (CASP), 2019.

For peer review only - http://bmjopen.bmj.com/site/about/guidelines.xhtml

23. Lewin S, Bohren M, Rashidian A, et al. Applying the GRADE-CERQual approach: making an overall CERQual assessment of confidence and creating a Summary of Qualitative Findings table. *Implement Sci* 2018;13
24. Harden A, Thomas J, Cargo M, et al. Cochrane Qualitative and Implementation Methods Group guidance series—paper 5: methods for integrating qualitative and implementation evidence within intervention effectiveness reviews. *Journal of clinical epidemiology* 2018;97:70-78.
25. Flemming K, Booth A, Garside R, et al. Qualitative evidence synthesis for complex interventions and guideline development: clarification of the purpose, designs and relevant methods. *BMJ global health* 2019;4(Suppl 1)
26. Thomas J, Harden A. Methods for the thematic synthesis of qualitative research in systematic reviews. *BMC medical research methodology* 2008;8(1):45.
27. Ritchie J. Spencer, l.(1994). Qualitative data analysis for applied policy research. *Bryman and Burgess (Eds), Analysing Qualitative Data* 1994:173-94.
28. Food, Administration D. Guidance for industry: patient-reported outcome measures: use in medical product development to support labeling claims. *Fed Regist* 2009;74(235):65132-33.
29. Peters M, Potter CM, Kelly L, et al. The long-term conditions questionnaire: conceptual framework and item development. *Patient related outcome measures* 2016;7:109.
30. Patrick DL, Burke LB, Gwaltney CJ, et al. Content validity—establishing and reporting the evidence in newly developed patient-reported outcomes (PRO) instruments for medical product evaluation: ISPOR PRO good research practices task force report: part 1—eliciting concepts for a new PRO instrument. *Value in Health* 2011;14(8):967-77.
31. Alam R, Figueiredo SM, Balvardi S, et al. Development of a patient-reported outcome measure of recovery after abdominal surgery: a hypothesized conceptual framework. *Surgical endoscopy* 2018;32(12):4874-85.

For peer review only

For peer review only - http://bmjopen.bmj.com/site/about/guidelines.xhtml

Figure legends:

Figure 1: The PROLLIT study

Figure 2: The three phases of developing a conceptual framework for patients requiring, undergoing or after undergoing reconstructive surgery for a lower limb condition that are outlined in this paper

For peer review only

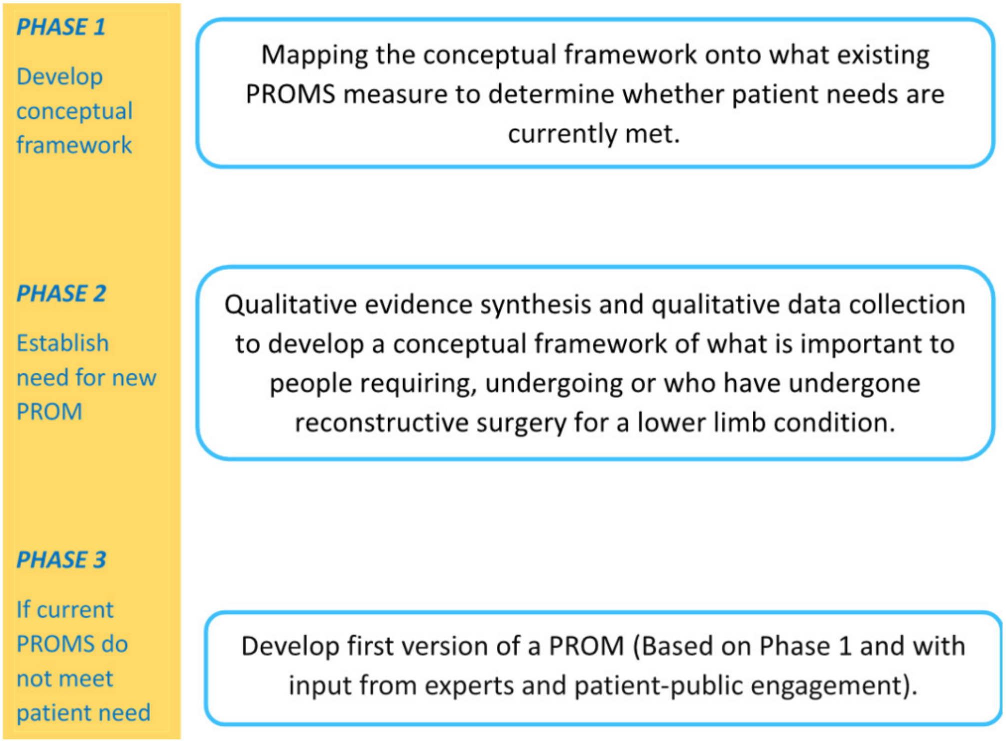

Figure 1. The PROLLIT study

90x90mm (300 x 300 DPI)

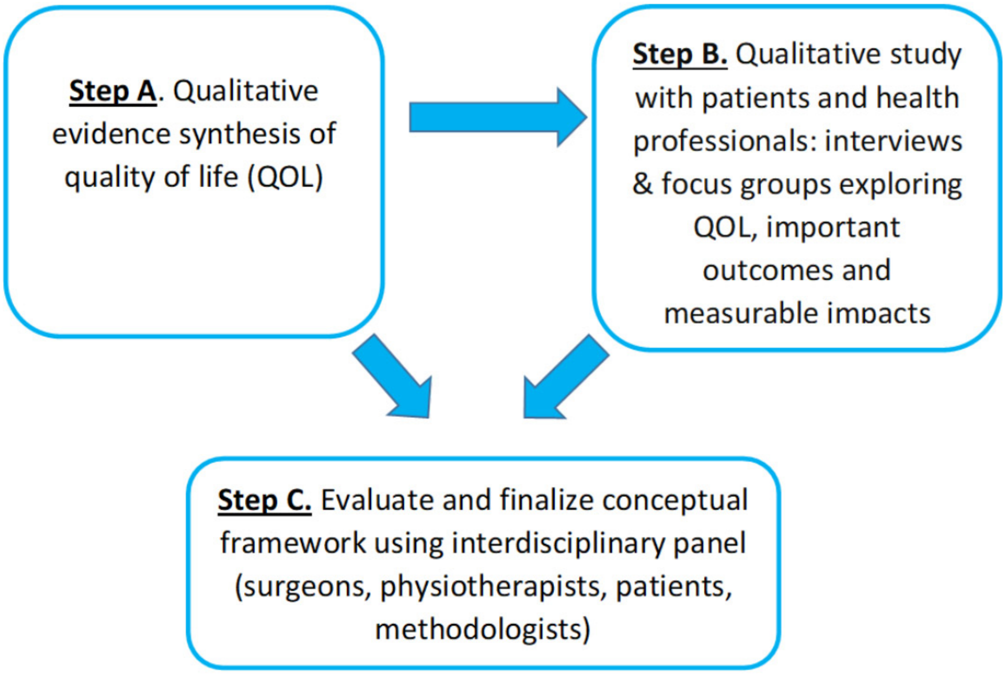

Figure 2. The three phases of developing a conceptual framework for patients requiring, undergoing or after undergoing reconstructive surgery for a lower limb condition that are outlined in this paper

90x90mm (300 x 300 DPI)

Database: Ovid MEDLINE(R) ALL <1946 to August 16, 2019>

19/08/2019

DRAFT Search Strategy:

----

1 Ankle Fractures/ (1253)

2 Femoral Fractures/ (15922)

3 Tibial Fractures/ (14766)

4 1 or 2 or 3 (29498)

5 ((fractur$ or trauma$) adj3 (lower extremit$ or lower limb$)).ti,ab. (2992)

6 ((fractur$ or trauma$) adj3 (leg or legs or thigh$ or knee or knees or shin or shins or shinbone$ or foot or midfoot or hindfoot or feet or ankle or ankles or anklebone$ or pilon or heel or heels or heelbone$ or toe or toes)).ti,ab. (9355)

7 ((fractur$ or trauma$) adj3 (femur$ or femoral or tibia$ or fibula$ or patella$ or kneecap$ or knee cap$)).ti,ab. (33008)

8 ((fractur$ or trauma$) adj3 (metatarsal$ or metatarsus or metatarsi or tarsus or tarsi or tarsal or tarsals or cuneiform or cuboid or navicular or calcane$ or talus or astragalus or tali or bimalleol$ or malleol$ or trimalleol$)).ti,ab. (5249)

9 ((Pott$ or Cotton$) adj fractur$).ti,ab. (30)

10 or/5-9 (46979)

11 lower extremity/ or exp foot/ or knee/ or leg/ or thigh/ (146797)

12 leg injuries/ or ankle injuries/ or foot injuries/ or knee injuries/ (38409)

13 exp Leg Bones/ (92594)

14 exp Foot Bones/ (17063)

15 11 or 12 or 13 or 14 (273200)

16 (lower extremit$ or lower limb$).ti,ab. (94764)

17 (leg or legs or thigh$ or knee or knees or shin or shins or shinbone$ or foot or midfoot or hindfoot or feet or ankle or ankles or anklebone$ or pilon or heel or heels or heelbone$ or toe or toes).ti,ab. (396803)

18 (femur$ or femoral or tibia$ or fibula$ or patella$ or kneecap$ or knee cap$).ti,ab. (237589)

19 (metatarsal$ or metatarsus or metatarsi or tarsus or tarsi or tarsal or tarsals or cuneiform or cuboid or navicular or calcane$ or talus or astragalus or tali or bimalleol$ or malleol$ or trimalleol$).ti,ab. (36596)

20 or/15-19 (717215)

21 Fractures, Bone/ (62396)

22 20 and 21 (18698)

23 (fractur$ or trauma$).ti,ab. (546587)

24 15 and 23 (39512)

For peer review only

25 exp Fracture Dislocation/ (2087)

26 Fractures, Avulsion/ (117)

27 Fractures, Closed/ (3275)

28 Fractures, Comminuted/ (2413)

29 Fractures, Compression/ (2080)

30 Fractures, Malunited/ (1551)

31 Fractures, Multiple/ (114)

32 Fractures, Open/ (5278)

33 Fractures, Stress/ (3216)

34 exp Fractures, Ununited/ (10494)

35 Intra-Articular Fractures/ (1104)

36 Periprosthetic Fractures/ (941)

37 Fracture Healing/ (12723)

38 Crush Injuries/ (121)

39 (crush$ adj2 (injur$ or trauma$ or fractur$)).ti,ab. (3676)

40 or/25-39 (43086)

41 20 and 40 (18153)

42 exp Multiple Trauma/ (12521)

43 (polytrauma$ or poly-trauma$ or multitrauma$ or multi-trauma$).ti,ab. (4627)

44 ((complex$ or complicat$ or severe$ or severity or serious or major or multiple) adj2 (fractur$ or trauma$)).ti,ab. (36622)

45 or/42-44 (48056)

46 20 and 45 (7375)

47 4 or 10 or 22 or 24 or 41 or 46 (87320)

48 ((bone$ or fracture$) adj3 (heal or heals or healed or healing)).ti,ab. (21408)

49 ((bone$ or fracture$) adj3 (union$ or nonunion$ or non union$ or ununite$)).ti,ab. (8182)

50 ((bone$ or fracture$) adj3 (malunion$ or mal union$ or deform$)).ti,ab. (4430)

51 (osteomyelitis or ((bone$ or fracture$) adj3 infect$)).ti,ab. (28503)

52 exp Osteomyelitis/ (22216)

53 or/48-52 (66572)

54 20 and 53 (21088)

55 ((femur$ or femoral or tibia$ or fibula$ or patella$ or kneecap$ or knee cap$) adj3 (heal or heals or healed or healing)).ti,ab. (1389)

56 ((metatarsal$ or metatarsus or metatarsi or tarsus or tarsi or tarsal or tarsals or cuneiform or cuboid or navicular or calcane$ or talus or astragalus or tali or bimalleol$ or malleol$ or trimalleol$) adj3 (heal or heals or healed or healing)).ti,ab. (107)

For peer review only - http://bmjopen.bmj.com/site/about/guidelines.xhtml

57 ((femur$ or femoral or tibia$ or fibula$ or patella$ or kneecap$ or knee cap$) adj3 (union$ or nonunion$ or non union$ or ununite$)).ti,ab. (1699)

58 ((metatarsal$ or metatarsus or metatarsi or tarsus or tarsi or tarsal or tarsals or cuneiform or cuboid or navicular or calcane$ or talus or astragalus or tali or bimalleol$ or malleol$ or trimalleol$) adj3 (union$ or nonunion$ or non union$ or ununite$)).ti,ab. (151)

59 ((femur$ or femoral or tibia$ or fibula$ or patella$ or kneecap$ or knee cap$) adj3 (malunion$ or mal union$ or deform$)).ti,ab. (1822)

60 ((metatarsal$ or metatarsus or metatarsi or tarsus or tarsi or tarsal or tarsals or cuneiform or cuboid or navicular or calcane$ or talus or astragalus or tali or bimalleol$ or malleol$ or trimalleol$) adj3 (malunion$ or mal union$ or deform$)).ti,ab. (528)

61 ((femur$ or femoral or tibia$ or fibula$ or patella$ or kneecap$ or knee cap$) adj3 (osteomyelitis or infect$)).ti,ab. (1622)

62 ((metatarsal$ or metatarsus or metatarsi or tarsus or tarsi or tarsal or tarsals or cuneiform or cuboid or navicular or calcane$ or talus or astragalus or tali or bimalleol$ or malleol$ or trimalleol$) adj3 (osteomyelitis or infect$)).ti,ab. (360)

63 or/55-62 (7267)

64 Limb Salvage/ (3799)

65 ((limb or limbs) adj3 (restor$ or reconstruct$ or salvag$)).ti,ab. (8371)

66 Ilizarov Technique/ (1280)

67 External Fixators/ (5652)

68 Orthopedic Fixation Devices/ (4991)

69 Fracture Fixation/ (18000)

70 Bone Lengthening/ (2175)

71 Ilizarov.ti,ab. (2107)

72 (external adj2 (fixat$ or frame$ or cage$)).ti,ab. (9626)

73 (circular adj2 (fixat$ or frame$ or cage$)).ti,ab. (609)

74 Taylor Spatial Frame$.ti,ab. (194)

75 TSF.ti,ab. (1209)

76 True Lok Hex.ti,ab. (0)

77 TLHex.ti,ab. (0)

78 or/64-77 (43999)

79 20 and 78 (20689)

80 47 or 54 or 63 or 79 (108579)

81 exp qualitative research/ (47998)

82 "Surveys and Questionnaires"/ (433413)

83 Self Report/ (27877)

84 exp Attitude/ (546190)

For peer review only - http://bmjopen.bmj.com/site/about/guidelines.xhtml

85 Focus Groups/ (27342)

86 Ethnology/ (1581)

87 discourse analysis.mp. (1718)

88 content analysis.mp. (25061)

89 ethnographic research.mp. (887)

90 ethnological research.mp. (7)

91 constant comparative method.mp. (1463)

92 qualitative validity.mp. (19)

93 purposive sample.mp. (3194)

94 observational method$.mp. (713)

95 field stud$.mp. (14522)

96 theoretical sampl$.mp. (635)

97 phenomenology.mp. (8689)

98 phenomenological research.mp. (431)

99 life experience$.mp. (5101)

100 or/81-99 (959292)

101 interview$.mp. or interviews/ or Interviews as Topic/ (365560)

102 qualitative.mp. (224736)

103 or/101-102 (512625)

104 100 or 103 (1295594)

105 ethnograph$.mp. (10148)

106 phenomenol$.mp. (24867)

107 grounded theory.mp. (10833)

108 (grounded adj (theor$ or study or studies or research or analys?s)).mp. (10995)

109 (emic or etic or hermeneutic$ or heuristic$ or semiotic$).mp. (16188)

110 (data adj1 saturat$).tw. (1054)

111 participant observ$.tw. (4226)

112 (action research or cooperative inquir$ or co operative inquir$ or co-operative inquir$).mp. (3997)

113 (field adj (study or studies or research or observation$)).tw. (18880)

114 theoretical sampl$.mp. (635)

115 (purpos$ adj4 sampl$).mp. (13410)

116 (focus adj group$).mp. (47371)

117 (account or accounts or unstructured or open-ended or open ended or text$ or narrative$).mp. (603046)

118 (life world or life-world or conversation analys?s or personal experience$ or theoretical saturation).mp. (14630)

For peer review only - http://bmjopen.bmj.com/site/about/guidelines.xhtml

119 lived experience$.tw. (5075)

120 (theme$ or thematic).mp. (97515)

121 (observational adj (method$ or research or stud$)).mp. (142328)

122 questionnaire$.mp. (691305)

123 content analysis.mp. (25061)

124 thematic analysis.mp. (15722)

125 discourse analys?s.mp. (1756)

126 ((discourse$ or discurs$) adj3 analys?s).tw. (2062)

127 (constant adj (comparative or comparison)).mp. (4283)

128 narrative analys?s.mp. (1157)

129 or/105-128 (1573717)

130 104 or 129 (2205208)

131 80 and 130 (5967)

132 survey$.ti,ab. (598311)

133 (mixed method$ or multimethod$ or multi-method$ or multi method$).mp. (21354)

134 (patient$ adj5 (attitude$ or belief$ or believ$ or experienc$ or opinion$ or perceiv$ or perception$ or perspective$ or preference$ or view or views or viewpoint$)).ti,ab. (253656)

135 or/132-134 (841986)

136 80 and 135 (3107)

137 131 or 136 (8322)

138 exp animals/ not humans/ (4609653)

139 (rat or rats or mouse or mice or rodent or rodents or swine or porcine or murine or sheep or lamb or lambs or ewe or ewes or pig or pigs or piglet or piglets or sow or sows or rabbit or rabbits or kitten or kittens or dog or dogs or puppy or puppies or monkey or monkeys or horse or horses or foal or foals or equine or calf or calves or cattle or heifer or heifers or hamster or hamsters or chicken or chickens or livestock or goat or goats).ti. (2075994)

140 138 or 139 (4948773)

141 137 not 140 (7989)

142 limit 141 to english language (7071)

For peer review only

For peer review only - http://bmjopen.bmj.com/site/about/guidelines.xhtml

PRISMA-P 2015 Checklist

This checklist has been adapted for use with protocol submissions to *Systematic Reviews* from Table 3 in Moher D et al: Preferred reporting items for systematic review and meta-analysis protocols (PRISMA-P) 2015 statement. *Systematic Reviews* 2015 **4**:1

Section/topic	#	Checklist item	Information reported Yes	Information reported No	Line number(s)
**ADMINISTRATIVE INFORMATION**					
**Title**					
Identification	1a	Identify the report as a protocol of a systematic review	☒	☐	P4. L19-20
Update	1b	If the protocol is for an update of a previous systematic review, identify as such	☐	☒	
**Registration**	2	If registered, provide the name of the registry (e.g., PROSPERO) and registration number in the Abstract	☒	☐	P2 L11
**Authors**					
Contact	3a	Provide name, institutional affiliation, and e-mail address of all protocol authors; provide physical mailing address of corresponding author	☒	☐	P1. L11-20
Contributions	3b	Describe contributions of protocol authors and identify the guarantor of the review	☒	☐	P11. L13-19
**Amendments**	4	If the protocol represents an amendment of a previously completed or published protocol, identify as such and list changes; otherwise, state plan for documenting important protocol amendments	☐	☒	
**Support**					
Sources	5a	Indicate sources of financial or other support for the review	☒	☐	P10. L53
Sponsor	5b	Provide name for the review funder and/or sponsor	☒	☐	P10. L45. P10. L53
Role of sponsor/funder	5c	Describe roles of funder(s), sponsor(s), and/or institution(s), if any, in developing the protocol	☐	☒	
**INTRODUCTION**					
**Rationale**	6	Describe the rationale for the review in the context of what is already known	☒	☐	P3. L5-54
**Objectives**	7	Provide an explicit statement of the question(s) the review will address with reference to participants, interventions, comparators, and outcomes (PICO)	☒	☐	P5. L5-6
**METHODS**					

For peer review only

BioMed Central The Open Access Publisher

Section/topic	#	Checklist item	Information reported Yes	No	Line number(s)
**Eligibility criteria**	8	Specify the study characteristics (e.g., PICO, study design, setting, time frame) and report characteristics (e.g., years considered, language, publication status) to be used as criteria for eligibility for the review	☒	☐	P5. L20-60
**Information sources**	9	Describe all intended information sources (e.g., electronic databases, contact with study authors, trial registers, or other grey literature sources) with planned dates of coverage	☒	☐	P6. L 5-17
**Search strategy**	10	Present draft of search strategy to be used for at least one electronic database, including planned limits, such that it could be repeated	☒	☐	Supplementary file
STUDY RECORDS					
Data management	11a	Describe the mechanism(s) that will be used to manage records and data throughout the review	☒	☐	P6. L21
Selection process	11b	State the process that will be used for selecting studies (e.g., two independent reviewers) through each phase of the review (i.e., screening, eligibility, and inclusion in meta-analysis)	☒	☐	P6. L22-30
Data collection process	11c	Describe planned method of extracting data from reports (e.g., piloting forms, done independently, in duplicate), any processes for obtaining and confirming data from investigators	☒	☐	P6. L31-39
**Data items**	12	List and define all variables for which data will be sought (e.g., PICO items, funding sources), any pre-planned data assumptions and simplifications	☒	☐	P6. L36-38
**Outcomes and prioritization**	13	List and define all outcomes for which data will be sought, including prioritization of main and additional outcomes, with rationale	☒	☐	P6. L31-38
**Risk of bias in individual studies**	14	Describe anticipated methods for assessing risk of bias of individual studies, including whether this will be done at the outcome or study level, or both; state how this information will be used in data synthesis	☒	☐	P6. L44-50
DATA					
**Synthesis**	15a	Describe criteria under which study data will be quantitatively synthesized	☐	☒	
	15b	If data are appropriate for quantitative synthesis, describe planned summary measures, methods of handling data, and methods of combining data from studies, including any planned exploration of consistency (e.g., I^2, Kendall's tau)	☐	☒	
	15c	Describe any proposed additional analyses (e.g., sensitivity or subgroup analyses, meta-regression)	☐	☒	
	15d	If quantitative synthesis is not appropriate, describe the type of summary planned	☒	☐	P6/7. L55-26
**Meta-bias(es)**	16	Specify any planned assessment of meta-bias(es) (e.g., publication bias across studies, selective reporting within studies)	☐	☒	
**Confidence in cumulative evidence**	17	Describe how the strength of the body of evidence will be assessed (e.g., GRADE)	☒	☐	P6.L50

BioMed Central
The Open Access Publisher

BMJ Open

Quality of life following a lower limb reconstructive procedure: A protocol for the development of a conceptual framework

Journal:	*BMJ Open*
Manuscript ID	bmjopen-2020-040378.R2
Article Type:	Protocol
Date Submitted by the Author:	27-Oct-2020
Complete List of Authors:	Leggett, Heather; University of York, York Trials Unit<br>Scantlebury, Arabella; University of York, Centre for Reviews & Dissemination<br>Sharma, Hemant; Hull Royal Infirmary, Trauma and Orhtopadics<br>Hewitt, Catherine; The University of York, Health Sciences<br>Harden, Melissa; University of York, Centre for reviews and dissemination<br>McDaid, Catriona; University of York, York Trials Unit
**Primary Subject Heading**:	Qualitative research
Secondary Subject Heading:	Research methods
Keywords:	QUALITATIVE RESEARCH, Quality in health care < HEALTH SERVICES ADMINISTRATION & MANAGEMENT, Adult orthopaedics < ORTHOPAEDIC & TRAUMA SURGERY, Protocols & guidelines < HEALTH SERVICES ADMINISTRATION & MANAGEMENT

SCHOLARONE™
Manuscripts

I, the Submitting Author has the right to grant and does grant on behalf of all authors of the Work (as defined in the below author licence), an exclusive licence and/or a non-exclusive licence for contributions from authors who are: i) UK Crown employees; ii) where BMJ has agreed a CC-BY licence shall apply, and/or iii) in accordance with the terms applicable for US Federal Government officers or employees acting as part of their official duties; on a worldwide, perpetual, irrevocable, royalty-free basis to BMJ Publishing Group Ltd ("BMJ") its licensees and where the relevant Journal is co-owned by BMJ to the co-owners of the Journal, to publish the Work in this journal and any other BMJ products and to exploit all rights, as set out in our licence.

The Submitting Author accepts and understands that any supply made under these terms is made by BMJ to the Submitting Author unless you are acting as an employee on behalf of your employer or a postgraduate student of an affiliated institution which is paying any applicable article publishing charge ("APC") for Open Access articles. Where the Submitting Author wishes to make the Work available on an Open Access basis (and intends to pay the relevant APC), the terms of reuse of such Open Access shall be governed by a Creative Commons licence – details of these licences and which Creative Commons licence will apply to this Work are set out in our licence referred to above.

Other than as permitted in any relevant BMJ Author's Self Archiving Policies, I confirm this Work has not been accepted for publication elsewhere, is not being considered for publication elsewhere and does not duplicate material already published. I confirm all authors consent to publication of this Work and authorise the granting of this licence.

For peer review only

Quality of life following a lower limb reconstructive procedure: A protocol for the development of a conceptual framework

Leggett H[a*], Scantlebury A[a], Sharma H[b], Hewitt C[a], Harden M[c], McDaid C[a], the PROLLIT study collaborators.

a. York Trials Unit, Health Sciences, University of York, York, UK.

b. Department of Trauma & Orthopaedics, Hull University Teaching Hospitals, Hull, UK

c. Centre for Reviews and Dissemination, University of York, York, UK

** Corresponding author. heather.leggett@york.ac.uk*

Word count

4014

Abstract

Introduction

Lower limb conditions requiring reconstructive surgery can be either congenital or acquired from trauma, infection or other medical conditions. Patient reported outcome measures (PROMS) are often used by healthcare professionals to assess the impact of a patient's condition (and treatment) on quality of life. However, we are not aware of any measures developed specifically for people requiring lower limb reconstructive surgery. Consequently, it is not clear the extent to which current PROMs accurately and specifically measure the outcomes that are important to these patients.

Methods and analysis

The 'PROLLIT' (Patient Reported Outcome Measure for Lower Limb Reconstruction) involves three phases: to explore what is important to patients with regards to quality of life (phase 1), ascertain whether current measures adequately capture these experiences (phase 2) and if not begin the development of a new patient reported outcome measure (phase 3). The population of interest is people requiring, undergoing or after undergoing reconstructive surgery for a lower limb condition. In this paper we describe Phase 1, which aims to develop a conceptual framework to identify and map what is important to this group with regards to social interactions, employment, perceived health and quality of life after condition onset/injury and throughout recovery. The conceptual framework will be developed through three steps: (Step A) a qualitative evidence synthesis, (Step B) a qualitative study with patients and staff to explore patient's views and experiences of lower limb reconstructive surgery and (Step C) a roundtable discussion with key stakeholders where findings from Step A and Step B will be brought together and used to finalise the conceptual framework.

Ethics consideration and dissemination

Ethical approval has been granted for the qualitative data collection (Step B) from South Central Berkshire Research Ethics committee (REF:20/SC/0114). Findings from Steps A and B will be submitted for peer-reviewed publication in academic journals, and presented at academic conferences.

For peer review only - http://bmjopen.bmj.com/site/about/guidelines.xhtml

For peer review only

PROSPERO registration number: CRD42019139587

ISRCTN Registration number: ISRCTN75201623

Article Summary

Strengths and limitations of this study

- A conceptual framework will be developed to establish the areas of importance for patients' quality of life and will determine if there is a need to develop a new patient reported outcome measure.
- A multi-methods approach will be used to develop the conceptual framework, increasing the robustness of the study.
- Both patient and staff opinions on important areas relating to quality of life after a lower limb reconstruction will be sought which will offer a more encompassing and detailed exploration.
- Due to resource constraints only English language studies will be eligible for inclusion in the review, introducing the risk of language bias.
- There is a risk that some individuals may be more likely than others to agree to take part in the qualitative study limiting generalisability, though strategies are in place to mitigate against this.

For peer review only

For peer review only - http://bmjopen.bmj.com/site/about/guidelines.xhtml

Introduction

There are several different lower limb conditions requiring reconstructive surgery. These can be congenital or acquired from trauma, infection or other medical conditions. Conditions may include, but are not limited to bone loss, joint contracture, nonunion, malunion or bone deformity. Limb reconstruction is the orthopaedic subspecialty, treating sequel of complex trauma and congenital conditions; deformity correction, nonunion, malunion, bone infections, limb lengthening and complex trauma. Individuals with a lower limb condition often experience mobility impairments as well as pain, discomfort, sleep disturbance, anxiety and low mood which can affect their daily life and reduce their employment and social engagement opportunities[1] **.** Therefore, it is not surprising that the experience of living with a lower limb condition as well as the impact of initial trauma or condition onset and any follow-on consequences may have negative impacts on an individual's quality of life (QOL)[2]. Consequently, it is important to understand the experience of patients during and after reconstructive surgery for a lower limb condition in order to measure treatment effectiveness and to monitor and improve care, care management, resource management and health policy. Healthcare professionals and researchers regularly utilise patient-reported outcome measures (PROMs) with patients. They often include assessments of a patient's specific condition and the impact (of the condition and treatment) on QOL[3 4]. These are important aspects for knowing if, and how to improve quality of care for patients[4].

Commonly used outcome measures for this population are related to musculoskeletal function such as the Olerud-Molander Ankle Score (OMAS[5]), or generic measures such as the Disability Rating Index (DRI[6]), Short Form 12 Health Survey (SF-12[7]), The Short Form 36 Health Survey (SF-36[8]), EQ-5D-5L[9] or the Sickness Impact Profile[10] (SIP). These tools have been used in capturing and reporting the QOL of patients who have experienced lower limb reconstruction[11 12]. More recently, the Patient-Reported Outcomes Measurement Information System (PROMIS) Physical Function measure was validated in patients with an orthopaedic trauma to a lower extremity[12 13]. However, this measure did not include QOL assessment and was limited to patients with an isolated lower extremity fracture only (with either surgery or non-operative closed treatment). Research is currently also underway to develop a PROM specifically for children and adolescents with lower limb deformities[14] as well as for adult patients undergoing a lower limb circular frame fixation[15]. Whilst the work conducted by Antonios and colleagues[15] will be relevant to a subset of our population it excludes a large number of patients undergoing other reconstructive treatments. The focus of existing PROMS means it is not clear whether they accurately and specifically measure the outcomes that are important to patients requiring or undergoing reconstructive surgery for a lower limb condition. As such, the need for a measure specifically designed to capture outcomes important to those requiring or undergoing reconstructive surgery for a lower limb condition may be warranted and needs further exploration.

Despite increased awareness surrounding the value of including patients in the development of PROMS, patients are not being fully engaged[16] and there is concern over the extent to which existing PROMS used with patients who have experienced a lower limb condition are fit for the purpose of accurately capturing important patient experiences. We are not aware of any measures developed specifically for people with conditions requiring reconstructive surgery. This study, known as "PROLLIT" (Patient Reported Outcome Measure for Lower Limb Reconstruction) will address this evidence gap by adopting a three phase approach (Figure 1) to explore what is important to patients with regards to QOL (Phase 1); ascertain whether current measures adequately capture these experiences (Phase 2); and if not, to begin the development of a new measure (Phase 3).

For peer review only - http://bmjopen.bmj.com/site/about/guidelines.xhtml

In this protocol paper we outline Phase 1 of the PROLLIT study, which will involve developing a conceptual framework related to patients requiring, undergoing or after undergoing reconstructive surgery for a lower limb condition (Figure 2).

Insert Figure 1

Insert Figure 2.

For peer review only

Methods. Development of a Conceptual Framework

In phase 1, our three stepped approach for developing a conceptual framework involves a qualitative evidence synthesis (Step A), a qualitative study with health professionals and patients (Step B) and a roundtable discussion with key stakeholders (Step C; Figure 2). The conceptual framework will be developed to identify and map what is important to patients requiring, undergoing or after undergoing reconstructive surgery for a lower limb condition with regards to social interactions, employment, perceived health and QOL after injury and throughout recovery.

For readability, we describe the process of developing our conceptual framework in three steps. However, it is important to recognise that this is not a linear study and that framework development will be on-going and iterative with each 'step' of the study influencing the other and contributing to the framework's overall development. For example, the findings of the qualitative evidence synthesis (Step A) will result in an initial framework. These findings will then be used to develop the topic guides for the qualitative study (Step B), which, through semi-structured interviews will draw upon the direct experience of health professionals and patients to explore in more depth, and where appropriate add to the findings of the qualitative evidence synthesis. In step C the findings of the qualitative evidence synthesis and qualitative study will then be combined and taken to a roundtable discussion with key stakeholders, the purpose of which will be to refine and finalise our conceptual framework (Figure 2).

The conceptual framework will then be used to inform the wider PROLLIT study, where we will, depending on whether patient priorities are being met by existing PROMS, develop a new PROM for people requiring, undergoing or who have undergone reconstructive surgery for a lower limb condition (Figure 1).

Step A. Qualitative Evidence Synthesis

This qualitative evidence synthesis will explore what is important to patients with regards to QOL after experiencing a lower limb reconstructive procedure. The Cochrane Handbook and guidance for Qualitative Evidence Synthesis (QES) published by the Cochrane Qualitative and Implementation Methods Group[17] provides the methodological framework for the design and implementation of this review. This QES will only include studies of 'good quality' based on the developed inclusion and exclusion criteria. The QES protocol complies with the requirements of the Preferred Reporting

Items for Systematic Reviews and Meta-Analyses Protocol (PRISMA-P[18]) as far as possible as PRISMA focuses on reviews of interventions. The QES findings (and final report) will be reported according to ENTREQ guidelines[19], as appropriate for qualitative evidence syntheses.

Eligibility

Sample- Patients (Adults - 16+) requiring, undergoing or who have undergone limb reconstructive surgery for a lower limb condition (leg, ankle or foot). Conditions may include:

- Infection - A fracture fixation which becomes infected
- Nonunion - A fracture which does not heal
- Malunion / Deformity - A fracture which does not heal in correct position. Any acquired or congenital condition leading to bone deformity.
- Leg length discrepancy or bone loss
- Congenital lower limb deformities
- Joint contracture
- Lower limb injuries where further limb reconstruction is required.
- Poly-trauma patients (as long as one of the above criteria are met).

- Patients who have undergone a lower limb amputation will not be included

Time since condition onset/ reconstructive surgery: patients will be included at any time point after condition onset. For example, participants will be included if they are still in hospital, under hospital outpatient care regarding their lower limb condition/reconstructive surgery or if they have been discharged with no further follow-up.

Phenomenon of interest- Quality of life, including (but not limited to) social interactions, employment, perceived health and QOL after condition onset/injury and throughout recovery.

Design- We will include qualitative studies which use established qualitative methods such as interviews or focus groups and use established qualitative analytical approaches (e.g. thematic analysis, framework analysis, grounded theory) to analyse the findings. We will include mixed-methods research which meets the criteria of a qualitative study outlined above. We will exclude opinion pieces, commentaries, case studies, guidelines, audits, observational studies, RCTs and other experimental designs.

Evaluation- We will include studies reporting on patient's attitudes, perspectives and behaviours surrounding QOL in the broadest sense. This will encompass people's experiences of the condition (symptoms/pain/recovery), experiences of treatment, their physical, mental, emotional, social, daily and professional functioning (effect on working and any financial difficulties), as well as outcome expectations.

Research Type- Qualitative evidence synthesis.

For peer review only - http://bmjopen.bmj.com/site/about/guidelines.xhtml

For peer review only

Search strategy for the identification of relevant studies

The following databases will be searched: MEDLINE (Including: Epub Ahead of Print, In-Process & Other Non-Indexed Citations, MEDLINE Daily and MEDLINE, via Ovid), Embase (Ovid), PsycINFO (Ovid), and CINAHL Complete (Ebsco). A preliminary search strategy has been developed for Ovid MEDLINE in conjunction with an Information Specialist from the Centre for Reviews and Dissemination at the University of York (Supplementary file 1). The strategy in brief consists of a set of terms for our population of interest combined using the Boolean operator AND, with a set of terms covering the research methods of interest (qualitative and mixed methods). The search terms relating to qualitative methods are based upon previous search strategies evaluated by Shaw et al[20]. This strategy will be adapted for the remaining databases. Searches will be run from database inception until present, and will be limited to publications in English. A manual search of the reference list of included studies will be undertaken to identify other studies meeting the inclusion criteria.

Selection of studies and data extraction

All results will be imported into EndNote reference management software (Clarivate Analytics (formerly Thomson Reuters), Philadelphia, PA, USA) and de-duplicated. Titles of records will be screened by a single researcher (task divided between three researchers) to remove obviously irrelevant records. Using the stated eligibility criteria, the abstract of each record included from the title screen stage will be independently reviewed by two researchers (task divided between three researchers). The full text of each record included from the abstract screen stage will then be independently screened by two researchers, against the eligibility criteria (task divided between three researchers). Discrepancies will be resolved by discussion with a third researcher.

Data extraction will be undertaken on the included studies using Excel for key study details. A data extraction template will be developed and piloted within the research team in order to summarise key information and persistent themes within studies. Key information will include author names, design or methods including method of data collection and analysis, participant characteristics and results. Results extracted will relate to patients' condition, treatments, outcomes, experiences, attitudes and thoughts surrounding QOL and other relevant outcomes using the NVIVO software. Data extraction will be undertaken by one researcher and checked by a second.

Quality assessment

Guidance by the Cochrane Qualitative and Implementation Methods Group recommends researchers assess methodological strengths and weaknesses[21]. In line with this guidance, the Critical Appraisal Skills Programme[22] checklist will be used to assess the included studies. Each article will be assessed independently by two researchers. Any difficulties in identifying each study's methodological quality will be resolved by discussing the paper in question with a third researcher. GRADE-CERQual[23] will be used to assess the strength of the review's findings.

Data analysis and synthesis

Data synthesis will be in line with the Cochrane Qualitative and Implementation Methods Group guidelines for qualitative evidence synthesis[24]. Key study characteristics including details of the population, setting, methods and study quality will be tabulated and described narratively. Thematic synthesis will be undertaken to analyse the qualitative data; data analysis will be facilitated by the

For peer review only - http://bmjopen.bmj.com/site/about/guidelines.xhtml

use of NVIVO. Thematic synthesis lends itself well to this research question and the type of data we anticipate finding since it allows for the synthesis and integration of both thin and more richly detailed data into descriptive and analytic themes[25]. The thematic synthesis will be based on the entirety of the 'results' section of each included study and will involve a three stage process[26]. Firstly, both verbatim comments (quotes) and author observations (author interpretations) from included studies will be coded. Secondly, the identification of common findings from individual studies will lead to the creation of researcher developed descriptive themes to describe these findings. The first two stages will be undertaken independently by three researchers and then discussed as a group. Thirdly, analytical themes will be developed to describe and interpret these themes, moving beyond the content of the original articles. For example, multiple instances of patient reported frustration, fatigue, depression and anxiety may be grouped together as a theme entitled 'Psychological Impact' and then further explored to understand the impact of this on QOL. This is an iterative process achieved through independent and group discussions of the implications of each theme.

After our initial thematic analysis, we will hold a roundtable discussion amongst the research team to develop a framework where we will identify the important areas related to QOL for patients requiring, undergoing or after undergoing reconstructive surgery for a lower limb condition that were identified in the synthesis. These findings will be used to inform topic guides for the qualitative data collection in Step B and will contribute to the development of the initial, hypothesised conceptual framework in step C (see Figure 2).

Step B. Qualitative Data Collection

Design

A qualitative study using semi-structured interviews and focus groups will be undertaken with patients and staff (orthopaedic clinicians, specialist nurses and physiotherapists) working with orthopaedic patients at five NHS hospitals in England. It is anticipated that including the perspectives of both staff and patients, from a range of NHS hospitals will provide a more encompassing and detailed exploration of important QOL related outcomes for patients.

Participants and recruitment (selection of participants)

A sampling frame will be developed through collaboration with lead clinicians from the participating sites. Patients will be those who meet the 'Sample' eligibility criteria outlined in Step A above. Patients will be identified and invited to participate by orthopaedic clinicians, specialist nurses, physiotherapists or research nurses. The five sites are orthopaedic departments of varying size, with different orthopaedic specialities and are situated throughout England to offer geographical spread. A purposive sampling strategy will be adopted to ensure maximum variation according to: age, gender, length of time since injury or condition onset, different lower limb conditions, trauma/condition severity and treatment received. Care will be taken to ensure that those who are requiring, undergoing or who have undergone reconstructive surgery for a lower limb condition, and have received care through the NHS at one of the participating sites are included. To achieve maximum variation we aim to recruit between 50-75 patient participants across the five sites (10-15 at each site) and 10 staff members (2 from each site), however analysis will stop or continue beyond this if saturation has/ has not been reached. Data collection will continue until saturation is reached

For peer review only - http://bmjopen.bmj.com/site/about/guidelines.xhtml

and no new information is gained in the interviews/focus groups with regards to factors and outcomes surrounding patients' QOL.

Staff (orthopaedic clinicians, specialist nurses and physiotherapists) will be invited to participate in the study by either direct contact from the researcher or through the lead contact at each site who will be identified through the PROLLIT study's PI's professional contacts. The anticipated start and end dates for data collection are November 2020-February 2021.

Procedure

Patients will be invited to take part in either a one-to-one interview with a researcher (virtual or via telephone) or a focus group with a researcher and a maximum of seven other participants. Individual semi-structured interviews or focus groups (approx. 4-8 people) will be undertaken with the patient participants and will be led by an experienced qualitative researcher. The interviews/focus groups will be arranged at a time most appropriate for the participants. A mix of focus groups and interviews for patients allows for flexibility in recruitment and scheduling the interviewers/focus groups. The group setting of the focus group may also provide an opportunity for patients to feel more relaxed and open, as well as enabling patients to bounce thoughts and discussion points off each other. Staff will be invited to take part in a one-to-one interview with the researcher either virtual or via telephone. Due to the limited time availability of working staff it will not be practical to arrange focus groups.

It is anticipated that interviews will last between 30 and 60 minutes and focus groups between 1 to 2 hours. Topic guides will be used to guide the interview/focus groups. These will be developed from a literature review of the area, our QES findings and the initial hypothesised conceptual framework developed from step A. The topic guides will be reviewed by experts in the field and our patient, public involvement group. The topic guide will use open-ended questions to explore key health-related QOL factors and other factors relevant to patients who have experienced a lower limb condition that requires or required reconstructive surgery.

Through the use of the topic guide and questioning, concept elicitation will be undertaken in the interview/focus group by asking the participants questions which explore their thoughts, attitudes and beliefs surrounding what is important to them with regards to QOL in relation to requiring, undergoing or after reconstructive surgery for a lower limb condition. Important factors may include physical, social and psychological well-being as well as job and lifestyle related factors. The staff will be asked to discuss, from their perspective, what they perceive to be important treatment outcomes and goals for patients.

Ethics and consent

We will ensure that participants have read the information sheet provided and have a full understanding of the study before consent is obtained. Participants will be invited to take part and will be reminded of their right to withdraw at any time. All participants (staff and patients) will be informed that participation is voluntary and that their involvement and responses will remain anonymous. Informed consent will be taken prior to each interview or focus group. Verbal consent will be obtained on an on-going basis during interviews/focus groups Participants will be reminded that the interviews/focus groups will be audio-recorded, quotations may be used and published, but that all identifiable information will be removed. All participants will be provided with a unique ID to maintain their anonymity.

For peer review only - http://bmjopen.bmj.com/site/about/guidelines.xhtml

Analysis

Data will be analysed at the University of York by qualitative researchers (AS, HL), who will adopt an iterative approach to data analysis, involving regular discussion between the research team. The qualitative data will be recorded and transcribed verbatim with data analysis facilitated by the use of NVIVO. Quotations will be used where appropriate to provide evidence for the conclusions drawn when reporting the study's findings.

We have chosen to analyse our data using Framework analysis[27] a method which allows for flexibility during the analysis process, as data can either be collected and then analysed, or analysed alongside data collection. The analysis will involve data being sifted, charted and sorted in accordance with key issues and themes. In this study, the focus will be on drawing out and grouping into key themes the important factors and outcomes relating to patients' self-reported QOL, as well as staff views on important treatment and outcome goals for patients. This will enable the research team to understand in greater depth, and from a psychological standpoint, the areas that are important to patients. To achieve this, our analysis will follow the five stages of framework analysis as outlined by Richie and Spencer[25]: 1. familiarisation; 2. identifying a thematic framework; 3. indexing; 4. charting; and 5. mapping and interpretation. In stage 1 the researchers will become familiarised with and immersed in the transcripts by reading them through multiple times. Following this, coding of the transcripts will begin by identifying emerging themes related to patient's QOL. Coding will be deductively based on a-priori themes from the QES findings where appropriate, whilst also inductively allowing for new themes to emerge which may influence the conceptual framework development. These will be influenced by and be complimentary to the findings from the QES. Multiple iterations of this step will enable the development of a thematic framework (stage 2). Indexing and charting (stage 3, 4) will begin whereby portions of the text that relate to a particular theme will be identified and charted according to the theme it corresponds to. Finally, in stage 5 mapping and interpretation involves analysis of the characteristics of each charted theme through graphical representation of the themes and how they relate to each other[27]. At the end of our analysis, the findings of the framework will be combined with those of the qualitative evidence synthesis (step B) and used to develop the conceptual framework (step C).

Step C. Conceptual Framework Development

An initial conceptual framework will be developed using findings from the qualitative evidence synthesis and qualitative study. As previously discussed, findings will then be combined by the research team and a 'hypothesised conceptual framework' will be taken to a roundtable discussion with key stakeholders. Key stakeholders will include members of the project advisory panel, patient and public involvement group and research team.

Methodological guidance whilst developing the conceptual framework will be sought from guidelines and conceptual framework method papers as required[28-31]. The knowledge and experience of the advisory panel and the patient and public involvement group will also be vital for the final refinement of the conceptual framework in step C and they will be invited to work closely alongside the research team at this stage. The final conceptual framework will represent what is important to patients with regards to social interactions, employment, perceived health and QOL after injury and throughout recovery after lower limb reconstructive surgery.

For peer review only - http://bmjopen.bmj.com/site/about/guidelines.xhtml

Advisory Panel

An Advisory Panel has been convened, consisting of orthopaedic surgeons, physiotherapists and methodologists to provide support, opinion and insight at key times during the project and to support recruitment to the study. The group will be contacted through a variety of methods such as emails, face-to-face meetings, telephone and video conferencing.

Patient and Public Involvement

A patient and public involvement group will be engaged throughout all stages of the study from design to reviewing the final conceptual framework. To maximise the diversity of the group we will aim to recruit people from a range of relevant patient support and clinical groups such as physiotherapy groups at each of the study sites. Patient and public involvement will include, but is not limited to, reviewing documentation for the qualitative study as well as sense checking the domains of importance identified from the qualitative evidence synthesis and the final conceptual framework. This will ensure that everything is appropriate and comprehensible from a patient's perspective. Patients will not be involved in the recruitment or conduct of the study. The results of the study will be disseminated to the patient and public involvement group via email or shared at a regular meeting.

Ethics and Dissemination

Only step B requires ethical approval which has been given favourable opinion from South Central – Berkshire Research Ethics committee (REF:20/SC/0114). All necessary local research governance approvals will be obtained at each of the five study sites prior to data collection. The interviews/focus groups will not impact on the care of the patients or the staff professionally. All participants will be provided with written information about the study and informed consent will be obtained. Step A is registered on the international prospective register of systematic reviews (PROSPERO) database (CRD number: CRD42019139587) and step B is registered on the International Standard Randomised Controlled Trial Number Registry (ISRCTN75201623). We intend to publish the QES findings and the findings from the qualitative study in a peer reviewed journal, disseminate at relevant conferences and provide feedback on the findings to participants who are interested and other patient groups and organisations.

Risks and Mitigations

Potential risks to the undertaking of this research pertain mainly to the recruitment of participants for Step B. Due to Covid 19 it may be harder to recruit patients due to reduced contact between hospital staff and patients which may make it difficult to achieve our desired sample size or to obtain a representative sample of patients. To mitigate this the researchers will closely monitor recruitment and if necessary ask those identifying patients to actively recruit certain types of patients. To mitigate the risk of Covid 19 to participants and researchers all interviews and focus groups will now be conducted either virtually via an online platform or via the telephone.

For peer review only

Acknowledgements

The authors would like to thank the advisory panel and the advisory patient and public involvement group who provided insight into defining the population of interest, refining the study design and providing feedback on key materials for step B.

Sponsorship

The study is being sponsored by The University of York

Indemnity

The University of York will provide legal liability cover for their employed staff and participants in the study. Non negligent harm will not be covered.

Funding

This project is funded through Research Capability funding from Hull University Teaching Hospitals NHS Trust.

Competing interests

Dr. McDaid reports grants from Hull University Teaching Hospitals NHS Trust, during the conduct of the study; grants from British Orthopaedic Association, outside the submitted work.

Dr. Sharma reports grants from Health & Technology Assessment, during the conduct of the study; grants from BBraun, personal fees from Biocomposites and personal fees from Orthofix, outside the submitted work.

Ms Harden, Dr Leggett, Dr Scantlebury and Professor Hewitt have nothing to disclose.

Contributors

CM, CH, HS were involved in the study conception. CM, HS, CH, HL and AS designed the study. MH developed the search strategy for the QES. HL drafted the manuscript with input from AS, CM, HS, MH and CH. The PROLLITT Collaborators provided methodological and clinical advice. All authors and the PROLLIT study collaborators reviewed and approved the final manuscript.

PROLLIT Study Collaborators

Joy Adamson, PhD (The University of York), Georgina Jones, PhD (Leeds Beckett University), Kim Cocks, PhD (KCStats Consultancy), Joel Gagnier, PhD (University of Michigan), Paul Harwood, MBChB (Leeds Teaching Hospitals NHS Trust), David Ferguson, MBChB (South Tees Hospitals, NHS Trust), Reggie Hamdy, MBChB (McGill University) and Nando Ferriera, PhD (Stellenbosch University).

For peer review only - http://bmjopen.bmj.com/site/about/guidelines.xhtml

References

1. Rees S, Tutton E, Achten J, et al. Patient experience of long-term recovery after open fracture of the lower limb: a qualitative study using interviews in a community setting. *BMJ open* 2019;9(10):e031261.
2. Giannoudis PV, Harwood PJ, Kontakis G, et al. Long-term quality of life in trauma patients following the full spectrum of tibial injury (fasciotomy, closed fracture, grade IIIB/IIIC open fracture and amputation). *Injury* 2009;40(2):213-19.
3. Black N. Patient reported outcome measures could help transform healthcare. *Bmj* 2013;346:f167.
4. Brédart A, Marrel A, Abetz-Webb L, et al. Interviewing to develop Patient-Reported Outcome (PRO) measures for clinical research: eliciting patients' experience. *Health and quality of life outcomes* 2014;12(1):15.
5. Olerud C, Molander H. A scoring scale for symptom evaluation after ankle fracture. *Archives of orthopaedic and traumatic surgery* 1984;103(3):190-94.
6. Salen BA, Nordemar R. The Disability Rating Index: an instrument for the assessment of disability in clinical settings. *Journal of clinical epidemiology* 1994;47(12):1423-35.
7. Ware Jr JE, Kosinski M, Keller SD. A 12-Item Short-Form Health Survey: construction of scales and preliminary tests of reliability and validity. *Medical care* 1996:220-33.
8. Ware JE. Scoring the SF-36. *SF-36 Health Survey: Manual and Interpretation Guide* 1993
9. Herdman M, Gudex C, Lloyd A, et al. Development and preliminary testing of the new five-level version of EQ-5D (EQ-5D-5L). *Quality of life research* 2011;20(10):1727-36.
10. Bergner M, Bobbitt RA, Kressel S, et al. The sickness impact profile: conceptual formulation and methodology for the development of a health status measure. *International Journal of Health Services* 1976;6(3):393-415.
11. Hoogendoorn J, van der Werken C. Grade III open tibial fractures: functional outcome and quality of life in amputees versus patients with successful reconstruction. *Injury* 2001;32(4):329-34.
12. MacKenzie EJ, Bosse MJ, Pollak AN, et al. Long-term persistence of disability following severe lower-limb trauma: Results of a seven-year follow-up. *JBJS* 2005;87(8):1801-09.
13. Rothrock NE, Kaat AJ, Vrahas MS, et al. Validation of PROMIS physical function instruments in patients with an orthopaedic trauma to a lower extremity. *Journal of orthopaedic trauma* 2019;33(8):377-83.
14. Chhina H, Klassen A, Kopec JA, et al. International multiphase mixed methods study protocol to develop a patient-reported outcome instrument for children and adolescents with lower limb deformities. *BMJ open* 2019;9(5):e027079.
15. Antonios T, Barker A, Ibrahim I, et al. A Systematic Review of Patient-reported Outcome Measures Used in Circular Frame Fixation. *Strategies in Trauma and Limb Reconstruction* 2019;14(1):34.
16. Wiering B, de Boer D, Delnoij D. Patient involvement in the development of patient-reported outcome measures: a scoping review. *Health Expectations* 2017;20(1):11-23.
17. Harris JL, Booth A, Cargo M, et al. Cochrane Qualitative and Implementation Methods Group guidance series—paper 2: methods for question formulation, searching, and protocol development for qualitative evidence synthesis. *Journal of clinical epidemiology* 2018;97:39-48.
18. Moher D, Shamseer L, Clarke M, et al. Preferred reporting items for systematic review and meta-analysis protocols (PRISMA-P) 2015 statement. *Systematic reviews* 2015;4(1):1.
19. Tong A, Flemming K, McInnes E, et al. Enhancing transparency in reporting the synthesis of qualitative research: ENTREQ. *BMC medical research methodology* 2012;12(1):181.
20. Shaw RL, Booth A, Sutton AJ, et al. Finding qualitative research: an evaluation of search strategies. *BMC Medical Research Methodology* 2004;4(1):5. doi: 10.1186/1471-2288-4-5
21. Noyes J, Booth A, Cargo M, et al. Cochrane Qualitative and Implementation Methods Group guidance series—paper 1: introduction. *Journal of clinical epidemiology* 2018;97:35-38.
22. Checklist CQ. Oxford, UK: Critical Appraisal Skills Programme (CASP), 2019.

For peer review only

For peer review only - http://bmjopen.bmj.com/site/about/guidelines.xhtml

23. Lewin S, Bohren M, Rashidian A, et al. Applying the GRADE-CERQual approach: making an overall CERQual assessment of confidence and creating a Summary of Qualitative Findings table. *Implement Sci* 2018;13
24. Harden A, Thomas J, Cargo M, et al. Cochrane Qualitative and Implementation Methods Group guidance series—paper 5: methods for integrating qualitative and implementation evidence within intervention effectiveness reviews. *Journal of clinical epidemiology* 2018;97:70-78.
25. Flemming K, Booth A, Garside R, et al. Qualitative evidence synthesis for complex interventions and guideline development: clarification of the purpose, designs and relevant methods. *BMJ global health* 2019;4(Suppl 1)
26. Thomas J, Harden A. Methods for the thematic synthesis of qualitative research in systematic reviews. *BMC medical research methodology* 2008;8(1):45.
27. Ritchie J. Spencer, l.(1994). Qualitative data analysis for applied policy research. *Bryman and Burgess (Eds), Analysing Qualitative Data* 1994:173-94.
28. Food, Administration D. Guidance for industry: patient-reported outcome measures: use in medical product development to support labeling claims. *Fed Regist* 2009;74(235):65132-33.
29. Peters M, Potter CM, Kelly L, et al. The long-term conditions questionnaire: conceptual framework and item development. *Patient related outcome measures* 2016;7:109.
30. Patrick DL, Burke LB, Gwaltney CJ, et al. Content validity—establishing and reporting the evidence in newly developed patient-reported outcomes (PRO) instruments for medical product evaluation: ISPOR PRO good research practices task force report: part 1—eliciting concepts for a new PRO instrument. *Value in Health* 2011;14(8):967-77.
31. Alam R, Figueiredo SM, Balvardi S, et al. Development of a patient-reported outcome measure of recovery after abdominal surgery: a hypothesized conceptual framework. *Surgical endoscopy* 2018;32(12):4874-85.

For peer review only

Figure legends:

Figure 1: The PROLLIT study

Figure 2: The three phases of developing a conceptual framework for patients requiring, undergoing or after undergoing reconstructive surgery for a lower limb condition that are outlined in this paper

For peer review only

For peer review only - http://bmjopen.bmj.com/site/about/guidelines.xhtml

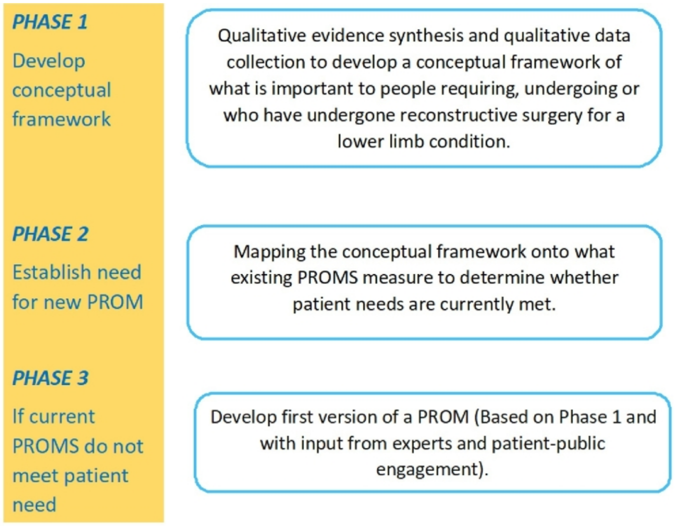

The PROLLIT study

186x101mm (300 x 300 DPI)

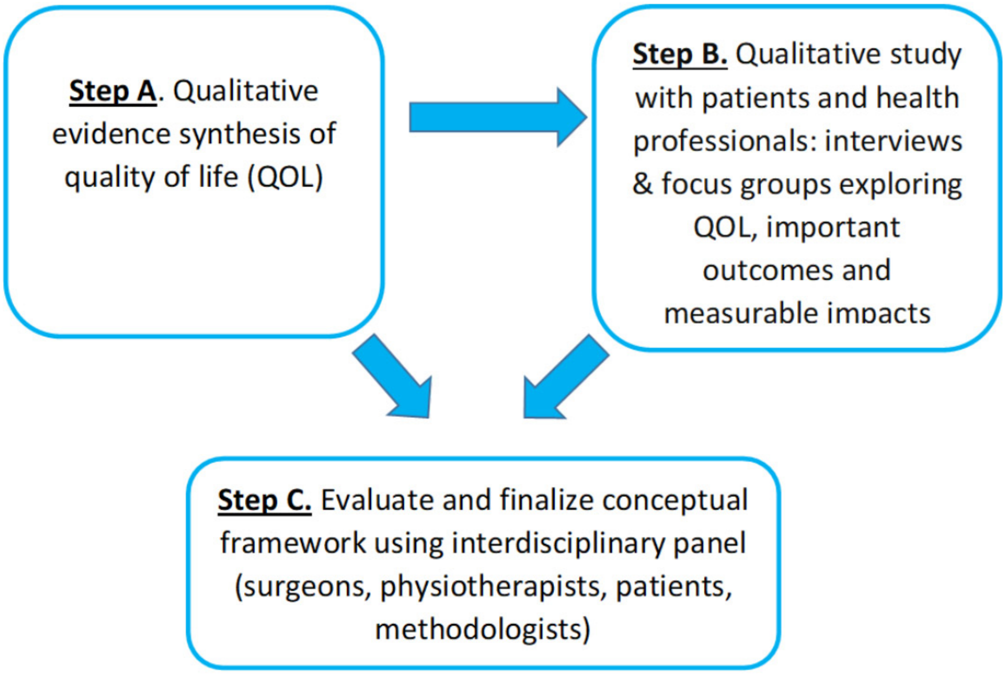

Figure 2. The three phases of developing a conceptual framework for patients requiring, undergoing or after undergoing reconstructive surgery for a lower limb condition that are outlined in this paper

90x90mm (300 x 300 DPI)

Database: Ovid MEDLINE(R) ALL <1946 to August 16, 2019>

19/08/2019

DRAFT Search Strategy:

----

1 Ankle Fractures/ (1253)

2 Femoral Fractures/ (15922)

3 Tibial Fractures/ (14766)

4 1 or 2 or 3 (29498)

5 ((fractur$ or trauma$) adj3 (lower extremit$ or lower limb$)).ti,ab. (2992)

6 ((fractur$ or trauma$) adj3 (leg or legs or thigh$ or knee or knees or shin or shins or shinbone$ or foot or midfoot or hindfoot or feet or ankle or ankles or anklebone$ or pilon or heel or heels or heelbone$ or toe or toes)).ti,ab. (9355)

7 ((fractur$ or trauma$) adj3 (femur$ or femoral or tibia$ or fibula$ or patella$ or kneecap$ or knee cap$)).ti,ab. (33008)

8 ((fractur$ or trauma$) adj3 (metatarsal$ or metatarsus or metatarsi or tarsus or tarsi or tarsal or tarsals or cuneiform or cuboid or navicular or calcane$ or talus or astragalus or tali or bimalleol$ or malleol$ or trimalleol$)).ti,ab. (5249)

9 ((Pott$ or Cotton$) adj fractur$).ti,ab. (30)

10 or/5-9 (46979)

11 lower extremity/ or exp foot/ or knee/ or leg/ or thigh/ (146797)

12 leg injuries/ or ankle injuries/ or foot injuries/ or knee injuries/ (38409)

13 exp Leg Bones/ (92594)

14 exp Foot Bones/ (17063)

15 11 or 12 or 13 or 14 (273200)

16 (lower extremit$ or lower limb$).ti,ab. (94764)

17 (leg or legs or thigh$ or knee or knees or shin or shins or shinbone$ or foot or midfoot or hindfoot or feet or ankle or ankles or anklebone$ or pilon or heel or heels or heelbone$ or toe or toes).ti,ab. (396803)

18 (femur$ or femoral or tibia$ or fibula$ or patella$ or kneecap$ or knee cap$).ti,ab. (237589)

19 (metatarsal$ or metatarsus or metatarsi or tarsus or tarsi or tarsal or tarsals or cuneiform or cuboid or navicular or calcane$ or talus or astragalus or tali or bimalleol$ or malleol$ or trimalleol$).ti,ab. (36596)

20 or/15-19 (717215)

21 Fractures, Bone/ (62396)

22 20 and 21 (18698)

23 (fractur$ or trauma$).ti,ab. (546587)

24 15 and 23 (39512)

For peer review only

25 exp Fracture Dislocation/ (2087)

26 Fractures, Avulsion/ (117)

27 Fractures, Closed/ (3275)

28 Fractures, Comminuted/ (2413)

29 Fractures, Compression/ (2080)

30 Fractures, Malunited/ (1551)

31 Fractures, Multiple/ (114)

32 Fractures, Open/ (5278)

33 Fractures, Stress/ (3216)

34 exp Fractures, Ununited/ (10494)

35 Intra-Articular Fractures/ (1104)

36 Periprosthetic Fractures/ (941)

37 Fracture Healing/ (12723)

38 Crush Injuries/ (121)

39 (crush$ adj2 (injur$ or trauma$ or fractur$)).ti,ab. (3676)

40 or/25-39 (43086)

41 20 and 40 (18153)

42 exp Multiple Trauma/ (12521)

43 (polytrauma$ or poly-trauma$ or multitrauma$ or multi-trauma$).ti,ab. (4627)

44 ((complex$ or complicat$ or severe$ or severity or serious or major or multiple) adj2 (fractur$ or trauma$)).ti,ab. (36622)

45 or/42-44 (48056)

46 20 and 45 (7375)

47 4 or 10 or 22 or 24 or 41 or 46 (87320)

48 ((bone$ or fracture$) adj3 (heal or heals or healed or healing)).ti,ab. (21408)

49 ((bone$ or fracture$) adj3 (union$ or nonunion$ or non union$ or ununite$)).ti,ab. (8182)

50 ((bone$ or fracture$) adj3 (malunion$ or mal union$ or deform$)).ti,ab. (4430)

51 (osteomyelitis or ((bone$ or fracture$) adj3 infect$)).ti,ab. (28503)

52 exp Osteomyelitis/ (22216)

53 or/48-52 (66572)

54 20 and 53 (21088)

55 ((femur$ or femoral or tibia$ or fibula$ or patella$ or kneecap$ or knee cap$) adj3 (heal or heals or healed or healing)).ti,ab. (1389)

56 ((metatarsal$ or metatarsus or metatarsi or tarsus or tarsi or tarsal or tarsals or cuneiform or cuboid or navicular or calcane$ or talus or astragalus or tali or bimalleol$ or malleol$ or trimalleol$) adj3 (heal or heals or healed or healing)).ti,ab. (107)

For peer review only - http://bmjopen.bmj.com/site/about/guidelines.xhtml

57 ((femur$ or femoral or tibia$ or fibula$ or patella$ or kneecap$ or knee cap$) adj3 (union$ or nonunion$ or non union$ or ununite$)).ti,ab. (1699)

58 ((metatarsal$ or metatarsus or metatarsi or tarsus or tarsi or tarsal or tarsals or cuneiform or cuboid or navicular or calcane$ or talus or astragalus or tali or bimalleol$ or malleol$ or trimalleol$) adj3 (union$ or nonunion$ or non union$ or ununite$)).ti,ab. (151)

59 ((femur$ or femoral or tibia$ or fibula$ or patella$ or kneecap$ or knee cap$) adj3 (malunion$ or mal union$ or deform$)).ti,ab. (1822)

60 ((metatarsal$ or metatarsus or metatarsi or tarsus or tarsi or tarsal or tarsals or cuneiform or cuboid or navicular or calcane$ or talus or astragalus or tali or bimalleol$ or malleol$ or trimalleol$) adj3 (malunion$ or mal union$ or deform$)).ti,ab. (528)

61 ((femur$ or femoral or tibia$ or fibula$ or patella$ or kneecap$ or knee cap$) adj3 (osteomyelitis or infect$)).ti,ab. (1622)

62 ((metatarsal$ or metatarsus or metatarsi or tarsus or tarsi or tarsal or tarsals or cuneiform or cuboid or navicular or calcane$ or talus or astragalus or tali or bimalleol$ or malleol$ or trimalleol$) adj3 (osteomyelitis or infect$)).ti,ab. (360)

63 or/55-62 (7267)

64 Limb Salvage/ (3799)

65 ((limb or limbs) adj3 (restor$ or reconstruct$ or salvag$)).ti,ab. (8371)

66 Ilizarov Technique/ (1280)

67 External Fixators/ (5652)

68 Orthopedic Fixation Devices/ (4991)

69 Fracture Fixation/ (18000)

70 Bone Lengthening/ (2175)

71 Ilizarov.ti,ab. (2107)

72 (external adj2 (fixat$ or frame$ or cage$)).ti,ab. (9626)

73 (circular adj2 (fixat$ or frame$ or cage$)).ti,ab. (609)

74 Taylor Spatial Frame$.ti,ab. (194)

75 TSF.ti,ab. (1209)

76 True Lok Hex.ti,ab. (0)

77 TLHex.ti,ab. (0)

78 or/64-77 (43999)

79 20 and 78 (20689)

80 47 or 54 or 63 or 79 (108579)

81 exp qualitative research/ (47998)

82 "Surveys and Questionnaires"/ (433413)

83 Self Report/ (27877)

84 exp Attitude/ (546190)

For peer review only - http://bmjopen.bmj.com/site/about/guidelines.xhtml

85 Focus Groups/ (27342)

86 Ethnology/ (1581)

87 discourse analysis.mp. (1718)

88 content analysis.mp. (25061)

89 ethnographic research.mp. (887)

90 ethnological research.mp. (7)

91 constant comparative method.mp. (1463)

92 qualitative validity.mp. (19)

93 purposive sample.mp. (3194)

94 observational method$.mp. (713)

95 field stud$.mp. (14522)

96 theoretical sampl$.mp. (635)

97 phenomenology.mp. (8689)

98 phenomenological research.mp. (431)

99 life experience$.mp. (5101)

100 or/81-99 (959292)

101 interview$.mp. or interviews/ or Interviews as Topic/ (365560)

102 qualitative.mp. (224736)

103 or/101-102 (512625)

104 100 or 103 (1295594)

105 ethnograph$.mp. (10148)

106 phenomenol$.mp. (24867)

107 grounded theory.mp. (10833)

108 (grounded adj (theor$ or study or studies or research or analys?s)).mp. (10995)

109 (emic or etic or hermeneutic$ or heuristic$ or semiotic$).mp. (16188)

110 (data adj1 saturat$).tw. (1054)

111 participant observ$.tw. (4226)

112 (action research or cooperative inquir$ or co operative inquir$ or co-operative inquir$).mp. (3997)

113 (field adj (study or studies or research or observation$)).tw. (18880)

114 theoretical sampl$.mp. (635)

115 (purpos$ adj4 sampl$).mp. (13410)

116 (focus adj group$).mp. (47371)

117 (account or accounts or unstructured or open-ended or open ended or text$ or narrative$).mp. (603046)

118 (life world or life-world or conversation analys?s or personal experience$ or theoretical saturation).mp. (14630)

For peer review only

For peer review only - http://bmjopen.bmj.com/site/about/guidelines.xhtml

119 lived experience$.tw. (5075)

120 (theme$ or thematic).mp. (97515)

121 (observational adj (method$ or research or stud$)).mp. (142328)

122 questionnaire$.mp. (691305)

123 content analysis.mp. (25061)

124 thematic analysis.mp. (15722)

125 discourse analys?s.mp. (1756)

126 ((discourse$ or discurs$) adj3 analys?s).tw. (2062)

127 (constant adj (comparative or comparison)).mp. (4283)

128 narrative analys?s.mp. (1157)

129 or/105-128 (1573717)

130 104 or 129 (2205208)

131 80 and 130 (5967)

132 survey$.ti,ab. (598311)

133 (mixed method$ or multimethod$ or multi-method$ or multi method$).mp. (21354)

134 (patient$ adj5 (attitude$ or belief$ or believ$ or experienc$ or opinion$ or perceiv$ or perception$ or perspective$ or preference$ or view or views or viewpoint$)).ti,ab. (253656)

135 or/132-134 (841986)

136 80 and 135 (3107)

137 131 or 136 (8322)

138 exp animals/ not humans/ (4609653)

139 (rat or rats or mouse or mice or rodent or rodents or swine or porcine or murine or sheep or lamb or lambs or ewe or ewes or pig or pigs or piglet or piglets or sow or sows or rabbit or rabbits or kitten or kittens or dog or dogs or puppy or puppies or monkey or monkeys or horse or horses or foal or foals or equine or calf or calves or cattle or heifer or heifers or hamster or hamsters or chicken or chickens or livestock or goat or goats).ti. (2075994)

140 138 or 139 (4948773)

141 137 not 140 (7989)

142 limit 141 to english language (7071)

For peer review only

For peer review only - http://bmjopen.bmj.com/site/about/guidelines.xhtml

PRISMA-P 2015 Checklist

This checklist has been adapted for use with protocol submissions to *Systematic Reviews* from Table 3 in Moher D et al: Preferred reporting items for systematic review and meta-analysis protocols (PRISMA-P) 2015 statement. *Systematic Reviews* 2015 **4**:1

Section/topic	#	Checklist item	Information reported Yes	Information reported No	Line number(s)
**ADMINISTRATIVE INFORMATION**					
**Title**					
Identification	1a	Identify the report as a protocol of a systematic review	☒	☐	P4. L19-20
Update	1b	If the protocol is for an update of a previous systematic review, identify as such	☐	☒	
**Registration**	2	If registered, provide the name of the registry (e.g., PROSPERO) and registration number in the Abstract	☒	☐	P2 L11
**Authors**					
Contact	3a	Provide name, institutional affiliation, and e-mail address of all protocol authors; provide physical mailing address of corresponding author	☒	☐	P1. L11-20
Contributions	3b	Describe contributions of protocol authors and identify the guarantor of the review	☒	☐	P11. L13-19
**Amendments**	4	If the protocol represents an amendment of a previously completed or published protocol, identify as such and list changes; otherwise, state plan for documenting important protocol amendments	☐	☒	
**Support**					
Sources	5a	Indicate sources of financial or other support for the review	☒	☐	P10. L53
Sponsor	5b	Provide name for the review funder and/or sponsor	☒	☐	P10. L45. P10. L53
Role of sponsor/funder	5c	Describe roles of funder(s), sponsor(s), and/or institution(s), if any, in developing the protocol	☐	☒	
**INTRODUCTION**					
**Rationale**	6	Describe the rationale for the review in the context of what is already known	☒	☐	P3. L5-54
**Objectives**	7	Provide an explicit statement of the question(s) the review will address with reference to participants, interventions, comparators, and outcomes (PICO)	☒	☐	P5. L5-6
**METHODS**					

For peer review only

BioMed Central The Open Access Publisher

Section/topic	#	Checklist item	Information reported Yes	No	Line number(s)
**Eligibility criteria**	8	Specify the study characteristics (e.g., PICO, study design, setting, time frame) and report characteristics (e.g., years considered, language, publication status) to be used as criteria for eligibility for the review	☒	☐	P5. L20-60
**Information sources**	9	Describe all intended information sources (e.g., electronic databases, contact with study authors, trial registers, or other grey literature sources) with planned dates of coverage	☒	☐	P6. L 5-17
**Search strategy**	10	Present draft of search strategy to be used for at least one electronic database, including planned limits, such that it could be repeated	☒	☐	Supplementary file
STUDY RECORDS					
Data management	11a	Describe the mechanism(s) that will be used to manage records and data throughout the review	☒	☐	P6. L21
Selection process	11b	State the process that will be used for selecting studies (e.g., two independent reviewers) through each phase of the review (i.e., screening, eligibility, and inclusion in meta-analysis)	☒	☐	P6. L22-30
Data collection process	11c	Describe planned method of extracting data from reports (e.g., piloting forms, done independently, in duplicate), any processes for obtaining and confirming data from investigators	☒	☐	P6. L31-39
**Data items**	12	List and define all variables for which data will be sought (e.g., PICO items, funding sources), any pre-planned data assumptions and simplifications	☒	☐	P6. L36-38
**Outcomes and prioritization**	13	List and define all outcomes for which data will be sought, including prioritization of main and additional outcomes, with rationale	☒	☐	P6. L31-38
**Risk of bias in individual studies**	14	Describe anticipated methods for assessing risk of bias of individual studies, including whether this will be done at the outcome or study level, or both; state how this information will be used in data synthesis	☒	☐	P6. L44-50
DATA					
**Synthesis**	15a	Describe criteria under which study data will be quantitatively synthesized	☐	☒	
	15b	If data are appropriate for quantitative synthesis, describe planned summary measures, methods of handling data, and methods of combining data from studies, including any planned exploration of consistency (e.g., I^2, Kendall's tau)	☐	☒	
	15c	Describe any proposed additional analyses (e.g., sensitivity or subgroup analyses, meta-regression)	☐	☒	
	15d	If quantitative synthesis is not appropriate, describe the type of summary planned	☒	☐	P6/7. L55-26
**Meta-bias(es)**	16	Specify any planned assessment of meta-bias(es) (e.g., publication bias across studies, selective reporting within studies)	☐	☒	
**Confidence in cumulative evidence**	17	Describe how the strength of the body of evidence will be assessed (e.g., GRADE)	☒	☐	P6.L50

BioMed Central
The Open Access Publisher
